# Robust regulation of transcription pausing in *Escherichia coli* by the ubiquitous elongation factor NusG

**DOI:** 10.1073/pnas.2221114120

**Published:** 2023-06-05

**Authors:** Alexander V. Yakhnin, Mikhail Bubunenko, Zachary F. Mandell, Lucyna Lubkowska, Sara Husher, Paul Babitzke, Mikhail Kashlev

**Affiliations:** ^a^RNA Biology Laboratory, Center for Cancer Research, National Cancer Institute, Frederick, MD 21702; ^b^Department of Biochemistry and Molecular Biology, Center for RNA Molecular Biology, Pennsylvania State University, University Park, PA 16802

**Keywords:** RNA polymerase pausing, NusG, RNET-seq, backtracking, translocation

## Abstract

Transcription elongation by multi-subunit RNA polymerases (RNAPs) is regulated by auxiliary factors in all organisms. NusG/Spt5 is the only universally conserved transcription elongation factor shared by all domains of life. NusG is a component of antitermination complexes controlling ribosomal RNA operons, an essential antipausing factor, and a transcription-translation coupling factor in *Escherichia coli*. We employed RNET-seq for genome-wide mapping of RNAP pause sites in wild-type and NusG-depleted cells. We demonstrate that NusG is a major antipausing factor that suppresses thousands of backtracked and nonbacktracked pauses across the *E. coli* genome. The NusG-suppressed pauses were enriched immediately downstream from the translation start codon but were also abundant elsewhere in open reading frames, small RNA genes, and antisense transcription units. This finding revealed a strong similarity of NusG to Spt5, which stimulates the elongation rate of many eukaryotic genes. We propose a model in which promoting forward translocation and/or stabilization of RNAP in the posttranslocation register by NusG results in suppression of pausing in *E. coli*.

## Significance

Pausing by RNA polymerase is essential for transcription in all domains of life. Little is known about how pausing is regulated by *trans*-acting factors and *cis*-acting signals in DNA/nascent RNA in vivo. Employing an advanced version of (nascent elongating transcript sequencing) NET-seq (RNET-seq), we found that the universally conserved transcription elongation factor NusG is a robust suppressor of backtracked and nonbacktracked pauses in *Escherichia coli*. Our in vivo work revealed a similarity of NusG to Spt5, its archaeal and eukaryotic homolog, which regulates the elongation rate of many eukaryotic genes.

**Fig. 1.**
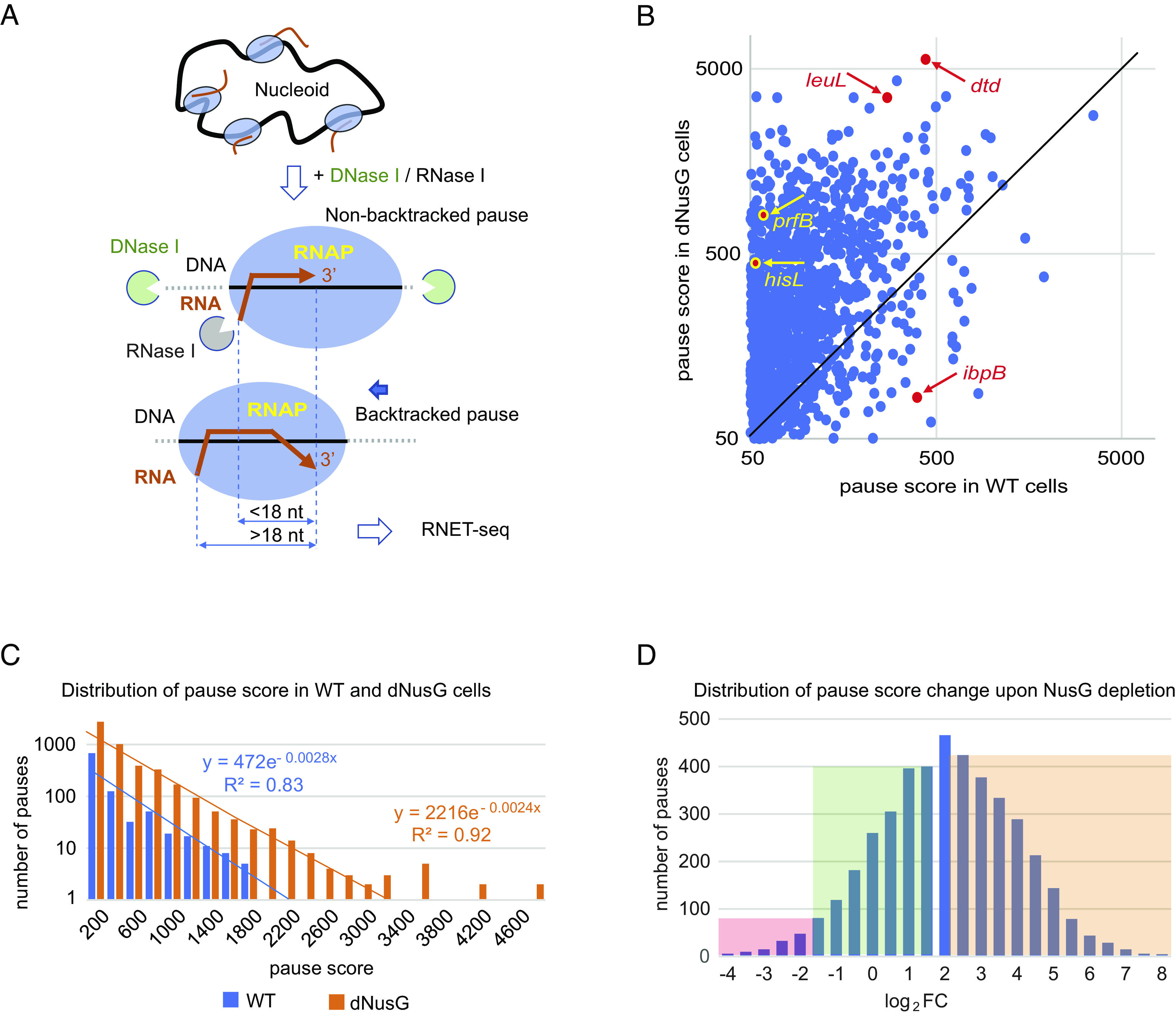
NusG is a general pause-suppressing factor in *E. coli.* (*A*) RNET-seq allowed determination of the RNAP translocation register in vivo by measuring the length of the nascent RNA reads protected by RNAP from RNase I digestion. Reads shorter than 18 nt and longer than 18 nt are generated from nonbacktracked and backtracked elongation complexes, respectively. (*B*) Pause score values in WT and dNusG *E. coli* cells. The black diagonal line (x = y) represents no change in score upon NusG depletion. Dots falling above or below the diagonal line correspond to pauses that increase or decrease in strength upon NusG depletion, respectively. The position of some pause sites that are discussed in the text is indicated. (*C*) Pauses are more abundant and have higher scores in dNusG cells than in WT cells. Pauses with a score value above 2,200 in WT cells and above 3,200 in dNusG cells are expected at a frequency of less than 1 per genome according to the negative exponential dependence of the number of pauses on pause score. These strongest pauses are listed in *SI Appendix*, Table S2. (*D*) Dramatically more pauses increase (log_2_FC>0) than decrease in strength (log_2_FC<0) upon NusG depletion. Shadowed in pink on the left are NusG-stimulated pauses that have a log_2_FC value below −1.5 (decrease in strength more than 2.8-fold). Shadowed in green are NusG-independent pauses that have −1.5 ≤ log_2_FC ≤ 1.5 values (unaffected in the range of 2.8-fold). Shadowed in orange on the right are NusG-suppressed pauses that have a log_2_FC value above 2 (increase in strength at least fourfold).

Transcription by multi-subunit RNA polymerases (RNAPs) is interrupted by random and programmed pauses (1). Numerous studies suggest that pausing results from temporary inactivation of the catalytic activity by misalignment of the 3′ end of the nascent transcript with the active site, which is caused by imperfect translocation of the enzyme along the DNA template and RNA product (2). Diverse sequence-specific molecular events such as holding RNAP in a pretranslocated or half-translocated register, misalignment of 3′-proximal RNA relative to the DNA template, hypertranslocation, and RNAP backtracking have been shown to cause transcription pauses in vitro (1–5). Pausing of *Escherichia coli* RNAP at a few sites from the above categories was characterized in detail in vitro, but only recent studies addressed pausing genome-wide by nascent elongating transcript sequencing (NET-seq). Genome-wide mapping of the nascent RNA 3′ ends at the pause sites revealed the consensus pause sequence in *E. coli* and enrichment of the pauses at translation initiation codons (6, 7). More recently, a modification of NET-seq was introduced that included digestion of cell-extracted elongation complexes with RNase (RNET-seq) (8). RNET-seq allowed determination of the RNAP translocation register of the paused complexes in vivo by measuring the length of nascent RNA reads protected by RNAP from RNase digestion (Fig. 1*A*). Similar to DNA footprinting of RNAP using digestion with exonuclease III (3), single base pair resolution of RNET-seq allowed assignment of the RNAP translocation register within paused elongation complexes as being pretranslocated, posttranslocated, or backtracked (8). However, the RNET-seq method could not distinguish half-translocated registers from posttranslocated registers. Thus, RNET-seq had the capability to reveal the prevailing molecular mechanism of RNAP pausing in vivo and to test existing pausing models.

Transcription elongation factors such as NusA, NusG, σ^70^ of RNAP, and the nascent RNA cleavage factors GreA and GreB dramatically affect RNAP pausing (5, 8, 9). The promoter recognition subunit of the *E. coli* RNAP holoenzyme (σ^70^) was shown to negatively regulate transcription elongation. Interaction of σ^70^ with a promoter-like sequence in the nontemplate DNA (ntDNA) strand during elongation promotes RNAP pausing (10–12). NusG is a transcription elongation factor universally conserved in all three domains of life, whereas NusA is not found in eukaryotes and archaeal NusA variants contain an incomplete domain complement of bacterial NusA (13, 14). First discovered as cofactors for lytic infection of *E. coli* by bacteriophage lambda, NusA and NusG were characterized as components of an antitermination complex that allows transcription of ribosomal RNA (rRNA) operons (15, 16). Initial testing of *E. coli* elongation factors in vitro suggested that NusA and NusG are negative and positive regulators of transcription elongation, respectively. NusA decreased the elongation rate and stimulated RNAP pausing and termination. In contrast, NusG accelerated the elongation rate and suppressed pausing (17). However, it was not known whether NusG suppressed pausing in vivo and what genes are regulated by NusG. Subsequent studies revealed different regulatory outcomes of NusG in other bacterial lineages outside of Proteobacteria. NusG stimulates RNAP pausing in *Bacillus subtilis* (phylum Firmicutes) both in vivo and in vitro by interaction with a sequence-specific motif in the ntDNA strand within the paused transcription bubble, a mechanism that shares some similarity with σ^70^-dependent pausing in *E. coli* (11, 18–20). NusG has also been shown to slow down transcription elongation in *Thermus thermophilus* (21). A group of specialized NusG paralogs are responsible for transcription of specific operons in response to a variety of sequence signals (22, 23). For example, recruitment of an NusG paralog RfaH to the *ops* sequence element in the ntDNA strand promotes pausing at the *ops* site, but suppresses pausing at all downstream pause sites, thereby enabling efficient transcription of long horizontally acquired operons in *E. coli* (22). The RNAP II elongation factor Spt5 is homologous to NusG, and it stimulates promoter-proximal pausing in eukaryotes. However, phosphorylation of Spt5 and its pause-promoting cofactor NELF by P-TEFb releases promoter-proximal pausing and converts Spt5 into a positive elongation factor (24). Collectively, the function of NusG proteins and their homologs are flexible, and their activity can be altered by external factors to regulate transcription elongation negatively or positively. As such, the genome-wide role of NusG/Spt5 proteins in transcription in each group of organisms can only be determined experimentally.

We assessed the genome-wide role of *E. coli* NusG in pausing in vivo by depleting this essential protein using a dCas9 *nusG* knockdown strategy (*SI Appendix*, *Materials and Methods* and Fig. S1). Using RNET-seq, we found that NusG is a general pause-suppressing factor in *E. coli* responsible for strong suppression of thousands of pauses genome-wide. We also found that the previously identified consensus pauses enriched at translation start codons are targets for NusG. NusG depletion reduces expression of genes for rRNA and ribosomal proteins, but increases expression of genes involved in the DNA damage response, suggesting that the role of NusG in genome stability occurs through resolution of conflicts between replication and transcription. In contrast to the numerous in vitro observations, NusG only mildly suppresses RNAP backtracking in vivo. We propose a model in which NusG suppresses pausing by promoting forward translocation or stabilizing RNAP in the posttranslocation register.

## Results

### Principles of RNET-Seq.

We previously developed combination of RNase footprinting of the transcripts with native elongating transcript sequencing (RNET-seq), an advanced version of NET-seq that enables mapping of paused RNAP molecules throughout bacterial genomes with single base pair resolution (8). In contrast to NET-seq, RNET-seq allows identification of the RNAP translocation register at each pause site in vivo (12, 20). This method includes coprecipitation of RNAP carrying the nascent RNA with chromosomal DNA, followed by treatment with RNase I that eliminates all released RNAs that are not strongly protected by RNA-binding proteins, and truncates all nascent RNAs to the short 3′-proximal regions protected by RNAP (RNAP footprint on nascent RNA, here and herein) (Fig. 1*A*). As shown previously, the length of the RNA footprint appeared to be a signature of the RNAP translocation register so that 16-17-nt, 18-nt, and >18-nt footprints correspond to the posttranslocated, pretranslocated, and backtracked register, respectively (8, 12, 20). Using RNET-seq, we recently revealed the global pause-stimulated activity of *B. subtilis* NusG (20). In this work, we applied RNET-seq to determine the impact of NusG on genome-wide transcription pausing in *E. coli*.

### Transcription Pauses in WT and NusG-Depleted (dNusG) Cells.

Our bioinformatic approaches for analysis of RNET-seq data are described in *SI Appendix*. Pause score is a metric that was developed to quantify the in vivo strength of a pause site based on RNET-seq data (20). NusG depletion dramatically increased the number of pauses (*SI Appendix*, Table S1) and generally increased the pause score (Fig. 1 *B* and *C*), which identified *E. coli* NusG as a global antipause factor in vivo. We observed a general negative exponential dependence between the number of pauses and score of pauses both in WT and dNusG cells (Fig. 1*C*). Interestingly, we identified several extremely strong pauses with score values above the *x* axis intercept of the negative exponential model, indicating that these pauses were expected at less than one occasion per genome (Fig. 1*C* and *SI Appendix*, Table S2).

Differential pause strength in WT and dNusG cells (log_2_FC-Pause-TPM) was calculated as fold change in pause score values normalized to transcript abundance (RNET-seq data, Dataset S5) upon NusG depletion for all pauses. Of note, 1971 pauses with log_2_FC-Pause-TPM values above 2 (fourfold increase) were considered NusG suppressed, whereas 1662 pauses with log_2_FC-Pause-TPM values between −1.5 and 1.5 (less than threefold impact) were considered NusG independent or unaffected (Fig. 1*D*). In addition, 201 pauses with log_2_FC-Pause-TPM values below −1.5 (~threefold decrease) were considered NusG stimulated. Due to their low number, strength, poor reproducibility among the biological replicates of WT RNET-seq data, and our inability to show stimulation by NusG in vitro, we did not characterize these pauses further (*SI Appendix*, Fig. S2 *B* and *D*). The average log_2_FC-Pause-TPM value was 2.4, indicating that NusG depletion resulted in more than a fivefold increase in pause score genome-wide (Fig. 1*D*).

**Fig. 2.**
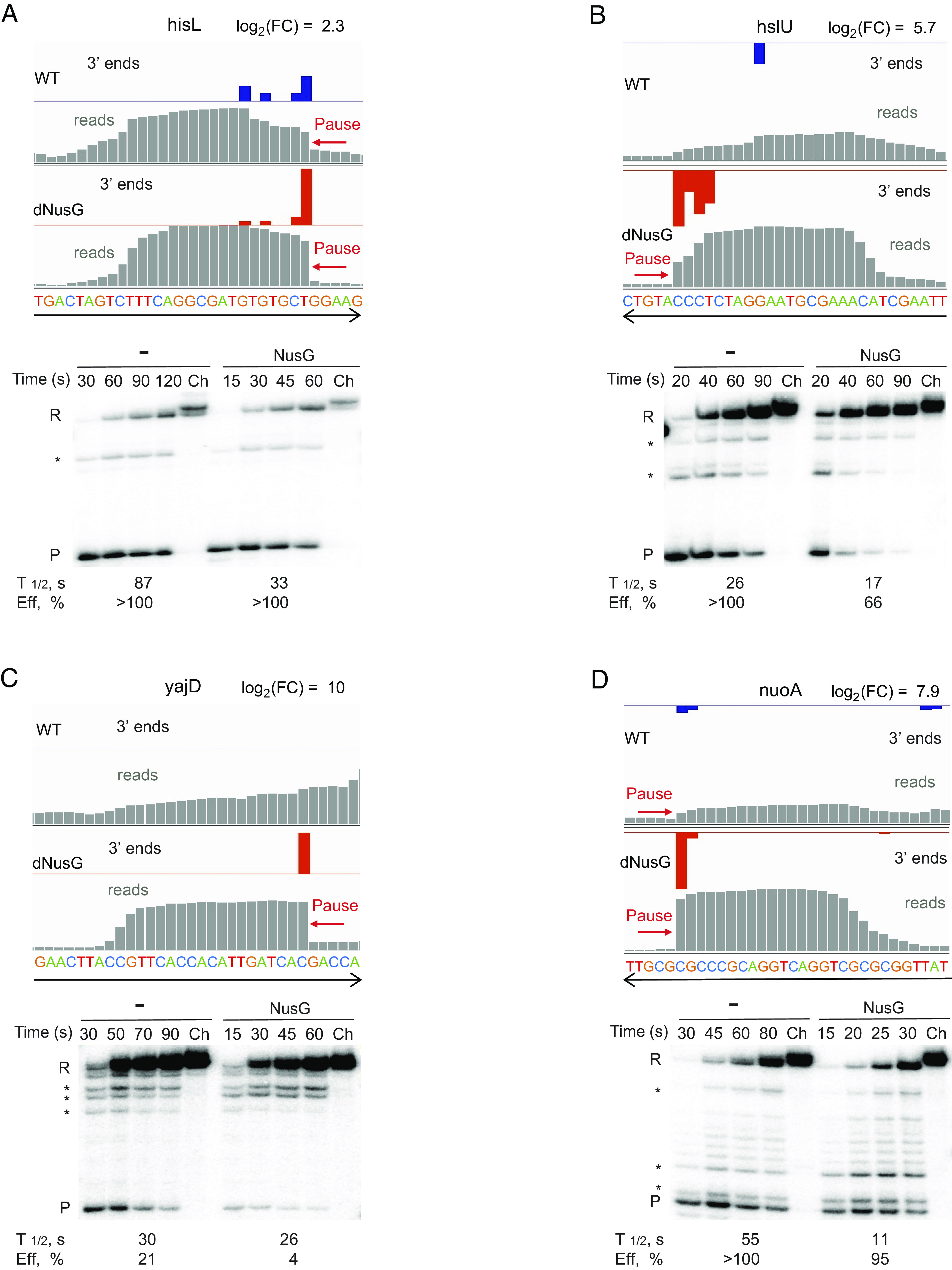
NusG-suppressed pause sites. Pause sites in the *hisL (A)*, *hslU (B)*, *yajD (C)*, and *nuoA (D)* genes in WT and dNusG cells identified by RNET-seq in vivo as they appear in the IGV browser (*Top* of each panel). Black arrows indicate the direction of transcription. Genome-aligned reads are in gray while mapped 3′ ends corresponding to the RNAP active site are in blue and red in WT and dNusG cells, respectively. Log_2_ values of fold change in pause score upon NusG depletion (log_2_FC) are indicated. Sequence around the pause site is indicated. The *Bottom* of each panel shows the results of a single-round in vitro transcription pause assays of the indicated templates in the absence and the presence of NusG (±NusG). Time points of the reaction are indicated above each lane. Ch, chase reactions. P, pause band; R, runoff transcript; *, minor pauses. The pause half-life (T_1/2_) and efficiency (Eff) are indicated below each set of lanes. The pausing efficiency exceeding 100% for some pauses results from the data fitting.

**Fig. 3.**
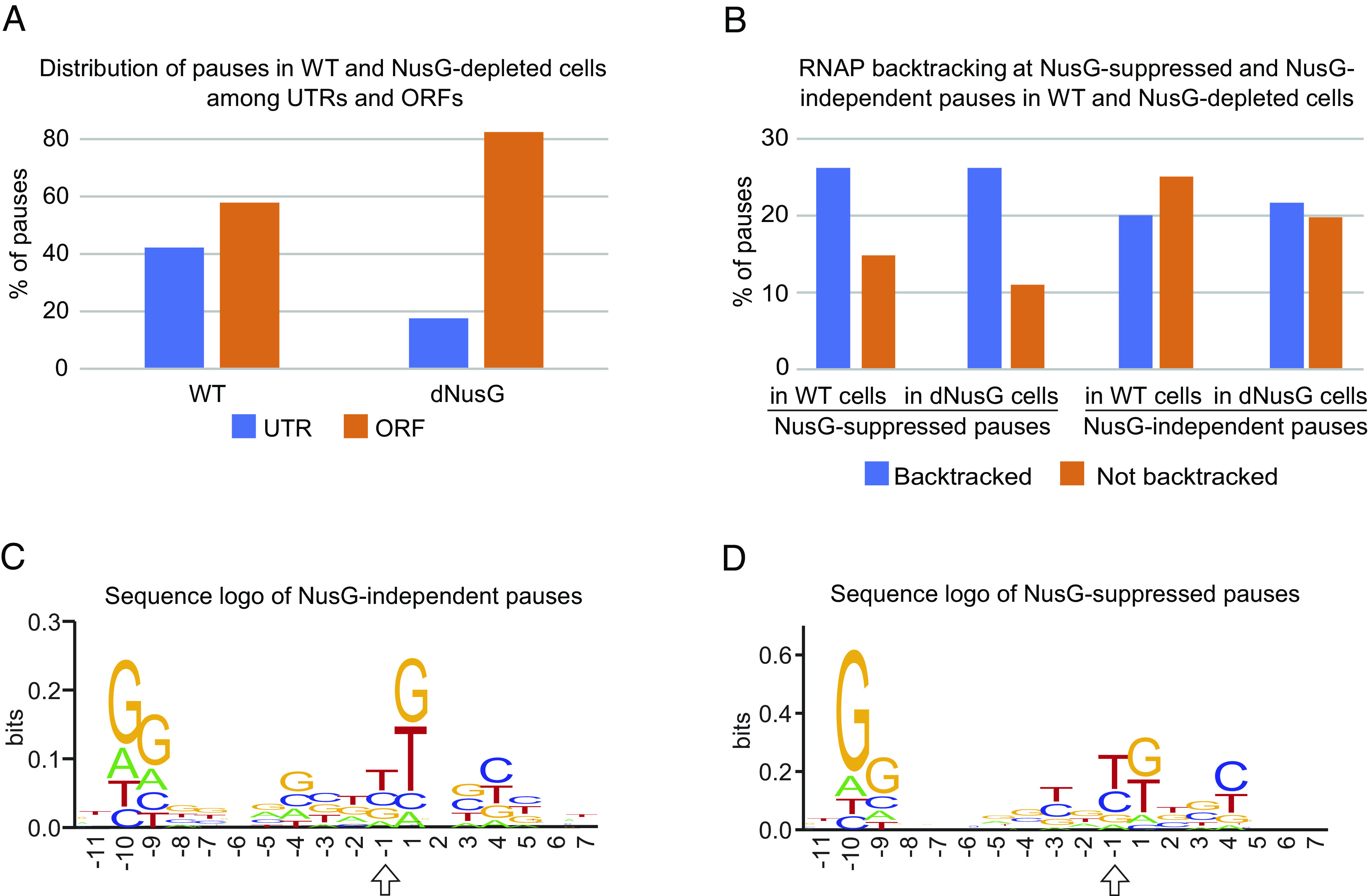
Genome-wide effect of NusG on pausing. (*A*) Distribution of strong pauses (score > 50) among untranslated (UTR) and protein-coding (ORF) sequences in WT and dNusG cells (Dataset S3). (*B*) Fraction of backtracked and nonbacktracked elongation complexes at NusG-suppressed and NusG-independent pauses in WT and dNusG cells (from Dataset S5). (*C* and *D*) Sequence logo of NusG-independent (*C*) and NusG-suppressed (*D*) pauses (from Dataset S5). Position −1 corresponds to the 3′ end of nascent RNA at the pause and is marked with an arrow.

Representative NusG-suppressed pause sites from our RNET-seq data are shown in Fig. 2 *A*–*D* and in *SI Appendix*, Fig. S2. The majority of pauses in dNusG cells (82%) and NusG-suppressed pauses (87%) were in open reading frames (ORFs) (Fig. 3*A* and *SI Appendix*, Fig. S3*A*). Eight percent of the NusG-suppressed pauses were located in antisense transcription units (*SI Appendix*, Fig. S3*B*). The average strength of pauses in antisense transcription units increased about twofold upon NusG depletion (*SI Appendix*, Fig. S4*A*). NusG-suppressed pauses were substantially enriched at the start codons, which matched the previously reported sharp increase of pausing in this region in general (*SI Appendix*, Fig. S5*A*) (6). In contrast, NusG-independent pauses were distributed more broadly in the region surrounding the start codons (±20 bp) (*SI Appendix*, Fig. S5*A*). The density of pauses increased at the beginning of ORFs in WT cells, whereas pauses were more evenly distributed along the ORFs in dNusG cells (*SI Appendix*, Fig. S6).

The NusG-suppressed pauses possessed a distinct sequence logo that was similar to the DNA logo of the previously reported *E. coli* “elemental” or “consensus” pause site (6–8). This logo consisted of −10G and −9G, a pyrimidine residue at the 3′ end of the nascent paused RNA (−1Y), and a +1G residue (Fig. 3*D*). However, the +1 position was equally occupied by G and T residues instead of a purine residue of the previously reported pause logo (6–8). In addition, the −10G residue was overrepresented in the logo compared to the −1Y and +1G residues (Fig. 3*D*).

### In Vitro Validation of NusG Effects on Pausing.

Inhibition of pausing by NusG was confirmed by in vitro transcription of several NusG-suppressed pause sites identified in vivo. Most frequently, NusG decreased both the pause efficiency (fraction of RNAP that pauses) and pause half-life (duration of pause) (Fig. 2 and *SI Appendix*, Fig. S2). However, the quantitative effect of NusG in vitro did not match the log_2_FC values calculated from the in vivo data. For example, at the intensively characterized *hisL* pause site, NusG suppressed pausing fivefold in vivo (log_2_FC = 2.3) but only threefold in vitro (Fig. 2*A*) (25, 26). We used a low concentration of a single NTP in vitro (most frequently GTP) to slow the rate of transcription to facilitate manual collection of elongation time points in a manageable time frame (25, 27). Transcription with low GTP limited in vitro pausing to a single position that is followed by a G base. In contrast, some of the in vivo pauses where the GTP was not limited contained multiple clustered 3′ ends (Fig. 2). The use of a low NTP concentration in vitro resulted in some additional pause sites that were undetectable or below the detection threshold that was set for the in vivo analysis (Fig. 2 and *SI Appendix*, Fig. S2).

We could not reproduce stimulation of pausing by NusG observed in vivo at several representative pause sites that we tested in vitro. Instead, we observed suppression of pausing at these sites in vitro (*SI Appendix*, Fig. S2 *B* and *D*). Presumably, NusG-stimulated pausing was an indirect consequence of NusG depletion and/or additional factors participate in NusG-mediated pause stimulation in vivo that are absent in vitro. In combination with poor reproducibility of the effect of NusG depletion on pausing at these sites in vivo, this class of pause site was not examined further.

### Effect of NusG on RNAP Backtracking In Vivo.

**Fig. 4.**
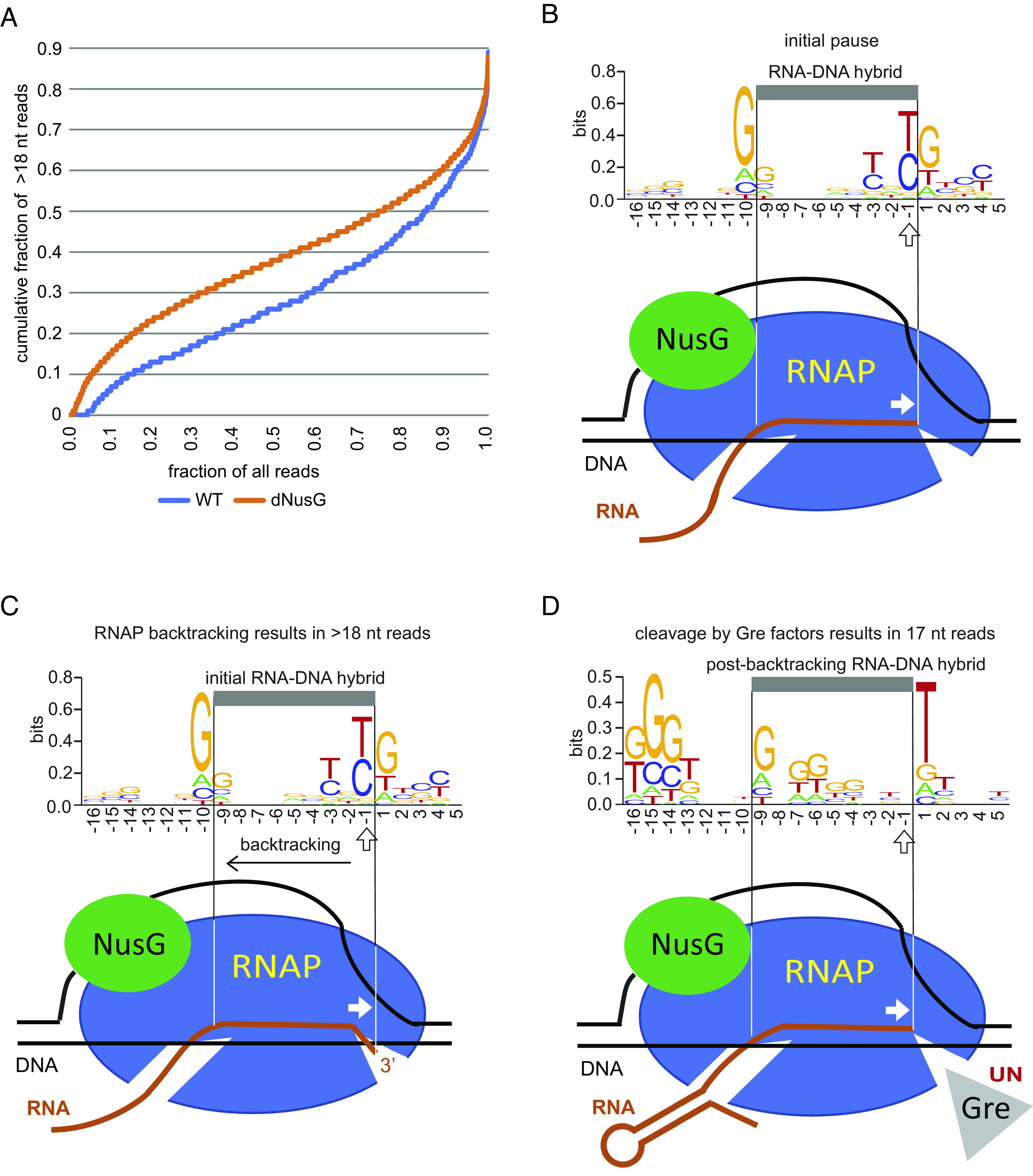
Backtracking in dNusG cells and processing of backtracked pauses by Gre factors in vivo. (*A*) Cumulative plot of RNET-seq reads longer than 18 nt (backtracked pauses) shows that RNAP is more prone to backtracking in dNusG cells compared to WT cells. (*B–D*) DNA logo analysis of RNAP pauses unique to dNusG cells. (*B*) The predicted position of initial nonbacktracked pauses containing the previously reported consensus −10G and −1T/C residues. Backtracking preserves this sequence signature of the initial pause before processing of these pauses by Gre factors (*C*). (*C* and *D*) The logo of backtracked pauses before (*C*) and after (*D*) cleavage stimulated by Gre factors; those pauses are enriched with >18-nt and 17-nt reads, respectively. The presence of a −10G residue in the logo of (*C*) and its shift to the −9G residue in (*D*) suggest that the G residue is favorable at the upstream edge of the RNA-DNA hybrid in both types of complexes. The cleavage of the >18-nt RNA stimulated by Gre produces the 17-nt reads, erases the sequence logo of the initial pause, and generates the new logo containing the −9G and +1T residues. In this scenario, the paused complexes share the 17-nt reads and −9G+1T sequence logo derived from backtracking followed by Gre-stimulated cleavage. The Gre-assisted cleavage sequence specificity (UN) is indicated next to Gre. The G-rich sequence at positions −16 to −14 of Gre-processed complexes (*D*) corresponds to the downstream arm of the RNA hairpin that blocks backtracking at this position (*SI Appendix*, Figs. S11 and S12). The G-rich sequence is barely visible in the logo of the backtracked complexes that are not processed by Gre factors (*C*) because the hairpin is missing or not uniformly positioned relative to the 3′ RNA in these complexes. Position −1 corresponds to the 3′ end of paused RNA and is marked with an arrow.

RNET-seq allows determination of the RNAP translocation state in vivo from the length of RNase-protected RNA footprints, which depends on the translocation register of RNAP. The read length distribution at each pause might indicate the presence of multiple interconvertible translocation states adopted by RNAP at each pause. We arbitrarily considered the pauses enriched in the fraction of >18-nt reads with frequencies above 0.5 and below 0.2 as backtracked and nonbacktracked pauses, respectively. The fraction of the long >18-nt backtracked reads was higher among pauses in dNusG cells compared to pauses in WT cells (Fig. 4*A*). However, the apparent increase in backtracking upon NusG depletion was derived from the analysis of 4,408 pause sites observed in dNusG but not in WT cells (Dataset S3 and *SI Appendix*, Table S1). Therefore, we were unable to evaluate the effect of NusG on backtracking at these sites. In contrast, the effect of NusG depletion on backtracking at the pauses shared by WT and dNusG cells was minimal. In fact, the average strength of the shared pauses increased more than twofold upon NusG depletion (*SI Appendix*, Fig. S5*C*) with only a small 2% increase of backtracking (*SI Appendix*, Fig. S5*D*). Thus, the fraction of backtracked pauses was nearly identical in WT and dNusG cells (Fig. 3*B*). This finding is inconsistent with the proposed general role of NusG in suppression of backtracking.

### Effect of NusG Depletion on the Transcriptome.

A combination of RNET-seq and RNA-seq data indicated that NusG depletion affected expression of numerous protein coding genes in *E. coli* (Dataset S7). Principal component analysis (PCA) revealed a different expression pattern in RNET-seq and RNA-seq data for WT and dNusG cells. Three biological replicates of RNET-seq and four biological replicates of RNA-seq formed 4 separate PCA clusters. Interestingly, a 70% variance in PC1 reflected the difference between RNA-seq and RNET-seq data, whereas a 16% variance in PC2 reflected the difference between WT and dNusG cells (*SI Appendix*, Fig. S7). Genes that increased or decreased expression upon NusG depletion at the greatest level in the RNA-seq and RNET-seq data are listed separately in *SI Appendix,* Tables S3 and S4. Both RNA-seq and RNET-seq data indicate that NusG depletion strongly increases expression of a subset of SOS regulon genes such as *recA* and *recN.* These genes are under the control of the LexA repressor and are typically up-regulated in response to DNA damage (Dataset S7 and *SI Appendix*, Table S3 and Fig. S8). The expression of genes involved in motility and saccharide transport decreased upon NusG depletion (*SI Appendix*, Table S4 and Fig. S8). Although the expression of many genes involved in biofilm formation were increased upon NusG depletion, expression of the stimulator of autoaggregation and biofilm formation Ag43 (gene *flu*) was decreased in these cells (*SI Appendix*, Table S3 and Fig. S8). According to RNET-seq data, NusG depletion increased the pause score in antisense transcripts and the relative abundance of antisense transcripts (*SI Appendix*, Fig. S4 *A* and *B*). Notably, changes in the abundance of released (terminated) RNA upon NusG depletion generally exceeded changes in the abundance of nascent RNA. For example, the average expression of nascent and total RNA increased 17-fold and 60-fold, respectively, among the top 50 genes whose expression increased in each of these groups. Similarly, the average expression of nascent (RNET-seq) and total (RNA-seq) RNA decreased 10-fold and 15-fold, respectively, among the top 50 genes whose expression decreased in each of these groups (Dataset S7). Note that *rac* prophage genes were excluded from this analysis since construction of the NusG depletion strain apparently resulted in prophage excision. Changes in gene expression in response to NusG depletion exhibited a low negative correlation with changes of the relative density of NusG-suppressed pauses; the correlation coefficient of linear regression was equal to −0.25 (*SI Appendix*, Fig. S4*C*). This result indicates that NusG-regulated pausing has only a minor contribution on the relative mRNA abundance in *E. coli*.

### Role of Upstream RNA Hairpins.

Nascent transcripts can form a secondary structure 10 to 13 nt upstream from the 3′ end of the RNA that dramatically stimulates pausing of bacterial RNAP both in vitro and in vivo (6, 20, 25, 26, 28). We evaluated the role of RNA hairpins in pausing in vitro at both NusG-suppressed and NusG-stimulated pause sites identified by RNET-seq using antisense oligonucleotides complementary to the upstream arm of the hairpin to interfere with the cotranscriptional RNA folding (*SI Appendix,* Table S5) (9, 17, 29). As expected, the previously identified hairpin-stimulated *hisL* pause had a high dependence on the RNA hairpin such that disruption of the hairpin by an antisense oligonucleotide decreased the pause half-life to a greater extent than the addition of NusG (Fig. 2*A* and *SI Appendix,* Fig. S9*A*). It is worth noting that the *hisL* pause site, which is the prototypical hairpin-stimulated pause, has a shorter than average hairpin to the 3′ end distance of 11 nt (29). The identified *yajD* and *ibpB* pauses exhibited more modest responses in which the antisense oligonucleotides affected the pause efficiency, pause half-life, or both, with the strongest effect on the *ibpB* pause efficiency. The antisense oligonucleotide had little effect on pausing at the *hslU* site, suggesting that this predicted hairpin does not function as an authentic pause hairpin (*SI Appendix,* Fig. S9 *B–D*). We conclude that pause-stimulating RNA hairpins are components of some NusG-suppressed sites, but that their impact varies depending on the site.

Several of the strongest pause sites characterized in vivo and in vitro, such as the *E. coli hisL* and the *B. subtilis trp* U144, *ribD* and *tlrB* pauses, contain pause hairpins that end with a G residue (9, 25, 30). The sequence logo of the pause sites identified in our current work revealed a conserved G-rich tract starting 14 nt upstream from the pause site at −1 (−14 position), which likely corresponds to the downstream arm of the hairpin. Notably, the G-rich tract was most prominent in the logo of the pauses enriched in 17 nt nonbacktracked reads but absent in the logo of their backtracked counterparts enriched in >18 nt reads (Fig. 4 *C* and *D* and *SI Appendix*, Fig. S10 *E* and *F*). Note that the 17 nt reads were, in part, the products of Gre factor–stimulated 3′ RNA cleavage of longer reads derived from backtracked complexes (Fig. 4 *B*–*D*). These results strongly suggest that the hairpins folded in the RNA exit channel of RNAP inhibit backtracking at sites where the hairpin to 3′ end distance was ≤14 nt. In this scenario, hairpins that form further upstream from the −14 position would allow limited backtracking until RNAP encounters the hairpin. Such backtracking would then lead to Gre factor–stimulated cleavage, thereby repositioning the 3′ end of the RNA in the active site of RNAP.

To test a role of the hairpins in the inhibition of backtracking, we selected three groups of 50 NusG-suppressed pause sites from Dataset S5, with a positive, negligible, or negative correlation between NusG-suppressed pausing and backtracking, respectively (Dataset S8). Sequence logos for each group revealed the presence of an upstream G-rich tract corresponding to the 3′ end of putative hairpins. Notably, this tract started at the −15 position for the sites with the positive correlation but was shifted to the −14 position for those with negligible or negative correlation between pausing and backtracking (*SI Appendix*, Fig. S11). This observation suggested that the single nucleotide difference in position of the hairpin relative to the 3′ end of the RNA distinguished between backtracked and nonbacktracked pauses, such that hairpins with a hairpin to the 3′ end distance of 13 nt inhibited backtracking, while those with a distance of 14 nt did not. Thus, the proximity of the hairpin to the 3′ end dictated whether paused RNAP was capable of backtracking. As such, pause sites with a hairpin to 3′ end distance ≥14 nt might constitute a specific group of pauses in which NusG prevents backtracking by suppressing the initial pause.

We used CLC Genomics Workbench 21.0.5 software to predict hairpins at 458 pause sequences that exhibit the strongest NusG-suppressed pausing (log_2_FC-Pause-TPM >3) (Dataset S9). The number of predicted hairpins was plotted as a function of the distance from the 3′ RNA end for the 458 pauses and for 458 scrambled derivatives of these sequences (*SI Appendix*, Fig. S12 and Dataset S9). This analysis revealed the presence of a palindromic sequence capable of forming an RNA hairpin at most of these sites. Importantly, the predicted hairpins were primarily positioned in a narrow 11 to 13 nt window upstream from the 3′ RNA end of the 458 pause sequences, but not of their scrambled counterparts. This result suggests that the NusG-suppressed pauses contain an RNA hairpin with a hairpin to 3′ end distance of 11 to 13 nt. We concluded that these hairpins contributed to the pausing efficiency and were responsible for inhibiting RNAP backtracking at these sites in WT and NusG-depleted cells.

### Role of NusG in Transcription Polarity.

Since the interaction of the NusG KOW domain with Rho contributes to a subset of Rho-dependent transcription termination events, it is likely that NusG depletion affects Rho activity (31). A polar effect caused by premature Rho-dependent termination is manifested by a gradual decrease of RNA coverage in the 5′-to-3′ direction in RNET-seq data. Depending on the translational context, NusG would either stimulate Rho-dependent termination by interacting with Rho or would inhibit Rho-dependent termination by bridging RNAP and a translating ribosome (31, 32). Thus, NusG depletion might increase transcriptional polarity by allowing more RNAP pausing and/or by uncoupling transcription and translation. Alternatively, the lack of NusG may reduce polarity because NusG is no longer available to interact with Rho. We looked for evidence of transcriptional polarity by examining two genes (*prfB* and *rho*) that are repressed by their own gene products (33, 34). Autoregulation of *prfB* depends on translation, whereas Rho-dependent termination directly autoregulates expression of *rho* itself. Inspection of our RNET-seq data from WT cells revealed evidence of polarity of *rho*, which contains strong Rho-dependent terminators in the 5′ UTR and in its ORF (*SI Appendix*, Fig. S13 *A* and *B*). [Although we are using 5′ UTR to indicate the 5′ leader regions, some of these leader regions contain short regulatory coding sequences (30).] However, only a slight increase in polarity was observed in dNusG cells, indicating that NusG-mediated coupling of transcription and translation and/or NusG–Rho interaction do not have a major influence on Rho-dependent termination of *rho*. Expression of *rho* is downregulated only twofold upon NusG depletion despite the presence of numerous NusG-suppressed pause sites that were not observed in WT cells (Dataset S7 and *SI Appendix*, Fig. S13 *A* and *B*). One possibility is that paused RNAPs that are subject to Rho-dependent termination are short lived and fall below our threshold for identification of pause sites.

The natural in-frame stop codon in the *prfB* transcript is targeted by the *prfB* gene product RF2, resulting in termination of translation inside its own gene in *E. coli* and other bacteria (33). However, the drop of 3′ end density toward the end of *prfB* is barely detectable in WT cells and is absent in dNusG cells. Moreover, no increase in polarity was observed downstream of the in-frame stop codon in our RNET-seq data in WT or dNusG cells (*SI Appendix*, Fig. S13 *C* and *D*). Instead, an NusG-suppressed pause site is located immediately upstream of the regulatory stop codon (*SI Appendix*, Fig. S13*E*). This arrangement is typical of regulatory pause sites in 5′ UTRs that provide additional time for sensing environmental signals and/or the recruitment of additional regulators. As such, specific sequences downstream of in-frame stop codons may dramatically affect transcriptional polarity. NusG depletion has a minor effect on genome-wide reduction of RNAP traffic in 5′-to-3′ direction for all *E. coli* ORFs, which we interpreted as a genome-wide manifestation of transcription polarity (*SI Appendix*, Fig. S13*F*). This result does not support global role of NusG in suppression of transcription polarity by coupling transcription and translation. It also argues against translation-mediated indirect impact of NusG depletion on transcription pausing.

## Discussion

### NusG Is a Robust Antipausing Factor in *E. coli*.

In this work, we identified a crucial role of the transcription elongation factor NusG in the suppression of RNAP pausing in UTRs, ORFs, and during antisense transcription, with substantial enrichment of these pause sites in close proximity to translation start codons. We recently revealed the global pause-stimulating activity of *B. subtilis* NusG (20). Therefore, the influence of this universally conserved elongation factor on pausing varies dramatically in bacterial lineages. NusG pause suppression in *E. coli* was confirmed for all 8 pause sites that we tested in vitro (Fig. 2 and *SI Appendix*, Fig. S2). We found that NusG-dependent pause suppression was stronger in vivo than in vitro, suggesting that additional protein factors and cellular conditions contribute to the antipausing activity of NusG in vivo. Matching these conditions in vitro is a major challenge considering that NusG is a component of multiprotein complexes associated with RNAP during elongation. These complexes can include NusA, NusB, NusE, SuhB, Rho, ribosomes, and/or other specialized transcription factors such as Mfd and UvrD that are transiently recruited to RNAP under certain conditions (31, 32, 35–37). It is also known that pausing is affected by the concentration of NTPs, divalent cations such as Mg^2+^, and DNA-bound proteins that interfere with forward translocation of RNAP (26, 38, 39). Some of these factors are likely to be responsible for quantitative difference between our in vitro and in vivo results.

### Uneven Distribution of NusG-Affected Pauses across the *E. coli* Genome.

Enrichment of pauses in UTRs of WT cells suggests that NusG could participate in regulating expression of target genes (Fig. 3*A*). On the other hand, the high number of NusG-suppressed pause sites in ORFs indicates that a common function of *E. coli* NusG is to suppress pausing in protein coding regions (*SI Appendix*, Table S1 and Fig. S3*A*). This finding is consistent with a previous ChIP-chip study showing that the occupancy of NusG on DNA is higher during transcription of ORFs compared to UTRs (40). Enrichment of strong antisense pauses suggests that they might participate in regulating antisense transcription; the fraction of antisense pauses is threefold higher among the 100 strongest pauses compared to all pauses in both WT and dNusG cells (*SI Appendix*, Fig. S3 *C–F*).

### Sequence Determinants of NusG-Suppressed Pausing.

**Fig. 5.**
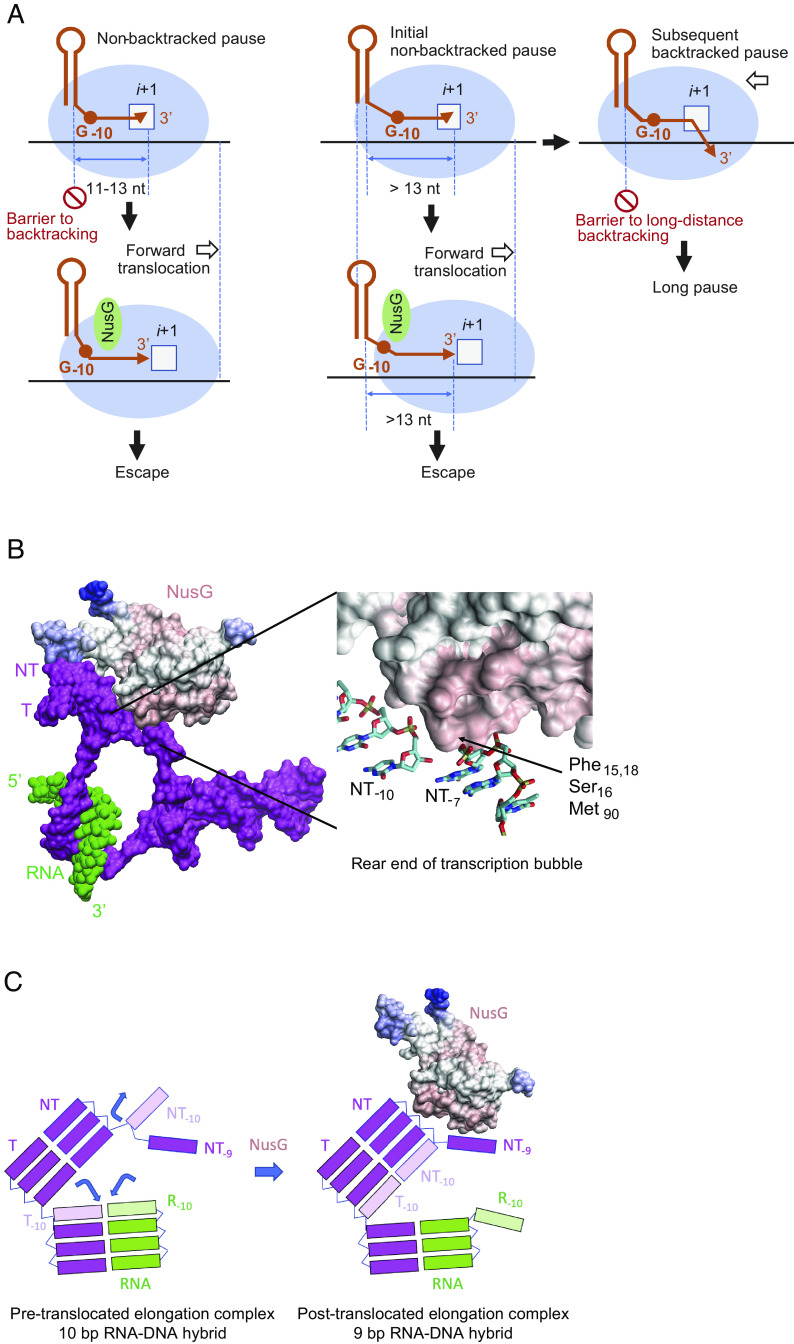
Model of NusG-suppressed backtracked and nonbacktracked pausing. (*A*) The scheme depicts the role of NusG, the −10G residue at the upstream edge of transcription bubble, and the RNA hairpin in nonbacktracked and backtracked pausing. The −10G residue interferes with forward translocation of RNAP at NusG-suppressed pause sites, and NusG counteracts the effect of the −10G residue. The RNA hairpin located 11 to 13 nt upstream from the pause inhibits backtracking at NusG-suppressed pause sites. See text for details. (*B*) *Left*, NusG binds to the upstream DNA duplex near the −11/−10 residues in the ntDNA strand (NT) according to the recent Cryo-EM structure PDB: 7PY0 (40). *Right*, magnified view of the NusG contacts with NT. (*C*) Binding of NusG to −10G may facilitate restoration of the DNA duplex at the upstream edge of the transcription bubble to promote forward translocation of RNAP by stabilization of dC:dG base pairing and/or limiting the ability of the −10G residue in NT to rotate away from its −10C partner in the template strand.

NusG-suppressed pause sites share sequence similarity to the previously identified consensus pause sequence that is enriched in pyrimidine residues at the 3′ end of the paused transcript, followed by a guanine residue in the ntDNA strand at position +1 (−1Y +1G motif). Another hallmark of the consensus sequence is a −10G residue (6), which is a logo feature of each class of pause site that we identified, but particularly for the NusG-suppressed pauses (Fig. 3 *C* and *D*). Previous studies showed that the −1C +1G sequence interfered with forward translocation of RNAP and proper alignment of the 3′ end of the RNA in the active site of RNAP (5, 8). The −10G residue is located at or near the upstream edge of the transcription bubble and has been shown to stabilize the pretranslocation state of RNAP, suggesting that NusG may counteract this negative effect on translocation by promoting forward translocation into the posttranslocation register (41). The recent Cryo-EM structure of RNAP with NusA and NusG showed that NusG binds to the upstream DNA duplex near the −11/−10 residues in the ntDNA strand (42) further supporting this idea. Binding of NusG to −10G may facilitate restoration of the DNA duplex at the upstream edge of the bubble to promote forward translocation of RNAP (Fig. 5 *A* and *B*). NusG may stabilize the dC:dG base pair or limit the ability of −10G in the ntDNA strand to rotate away from its −10C partner in the template strand. Importantly, the proposed stabilization of DNA:DNA base pairing at the −10 position of the bubble results in shortening of the RNA:DNA hybrid in RNAP to 9 base pairs, which has been shown to promote forward translocation in vitro and stabilize the catalytically competent posttranslocated register (Fig. 5*C*) (43). NusG may also have an allosteric effect on the active center of RNAP by tightening the DNA clamp made by the N-terminal domain of the β′ subunit (44). Mobility of the clamp depends on the switch ½, lid/rudder, and the gate loop domains in RNAP, which are near the upstream edge of the RNA-DNA hybrid. Tightening of the clamp depends on interaction of the lid and rudder domains with the DNA-DNA and RNA-DNA branch points in the bubble where NusG binds (45). By increasing the rigidity of this segment of nucleic acids, NusG may stabilize the clamp, reduce thermal fluctuations of the RNA:DNA hybrid in the catalytic cleft, and align the 3′ end of the nascent RNA with the template DNA strand to stimulate catalysis (41). Backtracking of RNAP requires melting of the DNA duplex at the upstream edge of the bubble with simultaneous reannealing of the RNA strand to the template DNA strand (43). NusG may suppress backtracking by preventing reannealing of the RNA and DNA strands at the upstream edge of the bubble (41).

### NusG and Backtracked Pausing.

The role of NusG in the suppression of backtracking has been shown in vitro, but this NusG function has never been confirmed for *E. coli* in vivo (17, 22, 41, 46, 47). Although backtracking and delayed forward translocation represent two mechanisms of transcription pausing (48), the mechanisms of pausing in vivo and the role of NusG in this process were unknown. Our RNET-seq analysis enabled detection of backtracked pauses in vivo by comparison of the fractions of long (backtracked) footprints of RNAP on nascent RNA at each pause site in the presence and absence of NusG. This analysis showed that NusG had a much stronger effect on pausing than on backtracking (*SI Appendix*, Fig. S14), suggesting that the pause-suppression activity of NusG is generally unrelated to backtracking. This apparent disagreement between the in vitro and in vivo results could indicate that pauses detected by RNET-seq are derived primarily from delayed or incomplete forward translocation as opposed to backtracking (48, 49). The relatively small fraction of backtracked pauses in dNusG cells could also be explained by robust cleavage activity of Gre factors present in both WT and dNusG cells. We previously showed that Gre factors efficiently suppress backtracking in vivo as was evident from RNET-seq data from a Δ*greA* Δ*greB* mutant (8, 12). DNA logo analysis revealed that NusG-suppressed backtracked and NusG-suppressed nonbacktracked pauses share the −10G −1Y +1G pause motif. The extended logo analysis also identified a G/C-rich sequence motif in both logos starting from the −14 or −15 position (Fig. 4*D* and *SI Appendix*, Fig. S11). This enrichment suggested the presence of a conserved hairpin upstream that includes this G residue at or near its base. The in vitro transcription analysis using antisense oligonucleotides confirmed the presence of pause hairpins for several NusG-suppressed pause sites (*SI Appendix*, Fig. S9). Our genome-wide analysis using CLC Genomics Workbench 21.0.5 software is consistent with a hairpin to 3′ end distance of 11 to 13 nt being a common component of NusG-suppressed pause sites (*SI Appendix*, Fig. S12). Notably, the conserved G residues ending at −15 in the logo of backtracked pauses are shifted 1 nt upstream relative to the logo of nonbacktracked pauses (*SI Appendix*, Fig. S11). An RNA hairpin that forms within the RNA exit channel of RNAP interferes with backtracking at the *hisL* pause site (50). We propose that RNA hairpins that form with a hairpin to 3′ end distance of 11 to 13 nt are responsible for inhibition of backtracking at the majority of NusG-suppressed pause sites in vivo. On the other hand, backtracking was detected at a subset of the pause sites without a predicted hairpin or with a hairpin to 3′ end distance >13 nt. The finding that a properly positioned RNA hairpin is a common feature of NusG-suppressed pausing may explain the minor impact of NusG on backtracking observed in vivo.

### Effects of NusG Depletion on the Transcriptome and the SOS Response.

The massive increase in RNAP pausing in dNusG cells likely results in frequent collisions between paused RNAP and the replication fork as was previously suggested (51, 52). The resulting DNA damage is likely responsible for the observed induction of DNA repair genes including the *lexA*-dependent branch of the SOS regulon in dNusG cells (*SI Appendix*, Table S3).

NusG is a component of a transcription antitermination complex during transcription of rRNA operons, and disruption of this complex is likely responsible for the observed decrease in rRNA transcription in dNusG cells (16, 35) (*SI Appendix*, Fig. S15*A*). Genes for ribosomal proteins are negatively regulated by transcription attenuation or translation repression feedback loops when transcription of rRNA operons decreases (53, 54). Not surprisingly, expression of genes involved in ribosome assembly and translation is also repressed in dNusG cells (*SI Appendix*, Table S4).

NusG-affected genes include several transcription regulators, which likely exacerbates misregulation of gene expression in dNusG cells. Indeed, changes in the abundance of released RNA generally exceed changes in the abundance of nascent RNA in dNusG cells (Dataset S7). Expression of genes involved in flagellum-dependent cell motility and the stimulator of autoaggregation and biofilm formation Ag43 (gene *flu*) is reduced in dNusG cells (*SI Appendix*, Table S4 and Fig. S8). Thus, natural repression of Ag43 by RfaH, an NusG paralog and competitor for RNAP occupancy, is likely exacerbated in dNusG cells (55).

### Role of NusG in Pausing in the Proximity of Translation Start Codons.

RNAP pausing at and downstream from the translation start codon may provide time for association of a NusG-bound 30S ribosomal subunit with the paused RNAP, thereby facilitating initiation of translation. The ribosome-assisted recruitment of NusG would result in the formation of a coupled transcription-translation complex containing RNAP and a 30S ribosomal subunit that is bridged by NusG.

We propose a model in which NusG-suppressed pauses promote establishment of the coupling of transcription and translation in *E. coli* near translation start sites by recruitment of NusG by an ATG-dependent mechanism. In turn, formation of the 30S-NusG-RNAP complex triggers RNAP escape from the pause as depicted in *SI Appendix*, Fig. S16. In the model, RNAP pausing at or shortly downstream from an ATG start codon facilitates binding of a 30S-NusG complex to RNAP at the translation initiation region, thereby synchronizing RNAP pause escape with ribosome binding to the nascent RNA. Similarly, the high density of NusG-suppressed pauses in ORFs may support transcription-translation coupling across the entire translated region, with NusG serving as the bridge between the ribosome and RNAP.

## Conclusions

This work identified *E. coli* NusG as a robust suppressor of transcription pausing. NusG affects pausing in all functional regions of the *E. coli* genome including UTRs, ORFs, and antisense sequences with a notable enrichment in the vicinity of translation start sites. We proposed a unified mechanism in which NusG promotes forward translocation and stabilizes RNAP in the posttranslocation register that in turn suppresses pausing. Suppression of pausing also suppressed RNAP backtracking at a subgroup of *E. coli* pause sites in which secondary structure in the nascent transcript does not prevent backtracking.

## Limitations of the Study

We showed that NusG is a global inhibitor of RNAP pausing in *E. coli* affecting transcription of many genes. Although NusG-depleted cells continued to grow at a normal rate when they were collected for RNET-seq, we cannot fully exclude the possibility of indirect consequences of depletion of this essential elongation factor. Since complete genome sequencing of the NusG depletion was not performed, we cannot exclude the possibility of suppressor mutations. However, our strain was propagated without induction of depletion that minimized accumulation of the suppressors. As such, the NusG depletion strain is not expected to accumulate mutations at a rate higher than that of any other cell lineage propagated without selection. We grew cells for RNET-seq at 37 °C, but we performed in vitro pausing assays at 23 °C to slow up the reaction so that we could collect time points to accurately measure pause half-lives. In our previous works, we did not observe strong dependence of NusG activity on temperature (18, 20). Our genome-wide analysis indicated a minor effect of NusG depletion on transcription polarity (*SI Appendix*, Fig. S13*F*). Therefore, the *prfB* gene that contains a natural in-frame stop codon that we used to exemplify this phenomenon may not be a general model for transcriptional polarity.

## Materials and Methods

Construction of the *nusG* knockdown strain, plasmids, oligonucleotides, and the bioinformatic pipelines for analysis of RNET-seq data are described in *SI Appendix*.

### *E. coli* Growth for RNET-Seq.

All strains were grown at 37 °C in LB medium supplemented with 25 µg/mL of kanamycin. Cells from a frozen stock culture were streaked on LB agar plates and incubated overnight. A single colony was used to inoculate 6 mL of LB, which was grown overnight in a rotary shaker. Two milliliters of an overnight culture of NB1246 (WT strain) were used to inoculate a 250-mL LB culture. Six milliliters of an overnight culture of NB1247 (NusG depletion strain) were used to inoculate a 250-mL LB culture supplemented with 25 µg/mL of kanamycin. NB1247 was grown at 37 °C for 1 h, and arabinose was added to a final concentration 0.4% to induce expression of *dcas9* for depletion of NusG. Cells were harvested during midexponential phase growth when the culture reached an OD_600_ value between 0.4 and 0.5.

### Preparation and Sequencing of RNET-Seq Libraries.

Collection and lysis of cells, purification of native transcription elongation complexes using Ni^2+^-NTA agarose, recovery of nascent RNA, and preparation of RNET-seq libraries were described in detail in our previous publication (20). Libraries were sequenced at the NIH Intramural Sequencing Center (Rockville) on an Illumina HiSeq platform using 50-nt paired-end sequencing.

### In Vitro Transcription.

DNA templates and proteins used for in vitro transcription are described in *SI Appendix*. The two-step single-round in vitro transcription reaction was performed in buffer containing 40 mM Tris–HCl (pH 8.0), 5 mM MgCl_2_, 0.1 mM EDTA, 4% trehalose, and 4 mM DTT as described previously (20). In the first step, halted elongation complexes containing a 29-nt transcript were formed for 5 min at 37 °C in a 6.4-µL reaction containing 60 to 100 nM DNA template, ATP and GTP (32 µM each), 0.8 µM UTP, 50 µg/mL acetylated BSA, 75 µg/mL *E. coli* RNAP holoenzyme, and 5 µCi of [α-^32^P]UTP at 37 °C (no CTP). RNAP was added from a 20× stock solution containing 1.5 mg/mL RNAP in 50% glycerol. The halted complexes were split in two 3.2 µL aliquots and diluted twofold with 1× transcription buffer containing 200 µg/mL acetylated BSA, 400 µg/mL heparin, and 200 mM KCl ±4 µM NusG. Elongation was resumed at 23 °C by the addition of 4.2 µL of all four NTPs in transcription buffer together with 50 µg/mL heparin and 100 mM KCl. The final NTP concentrations were 150 µM of ATP, CTP, and UTP and 10 µM of GTP. Performing transcription elongation at 23 °C and low GTP concentration slows down the transcription elongation rate and allows reliable collection of short-lived pause complexes manually. Two µM aliquots of the transcription elongation reaction were removed at various times. Transcription of the last aliquot (chase reaction) was continued for 10 min at 37 °C with 0.5 mM of each NTP. Transcription was stopped by the addition of an equal volume of the gel loading solution (20 mM EDTA [pH 8.0], 0.2% SDS, 0.05% bromophenol blue, and 0.05% xylene cyanol in formamide). Samples were fractionated through standard 5% sequencing gels. RNA bands were visualized with a phosphorimager and quantified using ImageQuant software (GE Healthcare Life Sciences).

## Supplementary Material

Appendix 01 (PDF)

Dataset S01 (XLSX)

Dataset S02 (XLSX)

Dataset S03 (XLSX)

Dataset S04 (XLSX)

Dataset S05 (XLSX)

Dataset S06 (XLSX)

Dataset S07 (XLSX)

Dataset S08 (XLSX)

Dataset S09 (XLSX)

## Data Availability

A total of 80 Gb of sequencing data have been deposited in the National Center for Biotechnology Information Sequence Read Archive (BioProject ID PRJNA884691) (56). All other study data are included in this article and *SI Appendix*.
