# Supplementary material for: Robust regulation of transcription pausing in Escherichia coli by the ubiquitous elongation factor NusG
Source: Proc Natl Acad Sci U S A. 2023 Jun 5;120(24):e2221114120. doi: 10.1073/pnas.2221114120 (PMC10268239; doi:10.1073/pnas.2221114120)
Supplement: Supplementary file 1 — Appendix 01 (PDF) [file pnas.2221114120.sapp.pdf]

PNAS

Supporting Information for
Robust regulation of transcription pausing in *E. coli* by the ubiquitous elongation factor NusG

Alexander V. Yakhnin[a], Mikhail Bubunenko[a], Zachary F. Mandell[b,c], Lucyna Lubkowska[a], Sara Husher[a], Paul Babitzke[b] and Mikhail Kashlev[a,*]

[a] RNA Biology Laboratory, Center for Cancer Research, NCI, Frederick, MD 21702

[b] Department of Biochemistry and Molecular Biology, Center for RNA Molecular Biology, Pennsylvania State University, University Park, PA 16802

[c] Present address: Department of Molecular Biology and Genetics and Department of Biology, Johns Hopkins University, Baltimore, MD 21205

* Corresponding Author: Mikhail Kashlev
Email: kashlevm@mail.nih.gov

This PDF file includes:

Supporting text
Figures S1 to S18
Tables S1 to S10
Legends for Datasets S1 to S9
SI References

Other supporting materials for this manuscript include the following:

Datasets S1 to S9

Supporting Information Text

Depletion of an essential protein in *E. coli*. In contrast to *B. subtilis*, NusG is an essential protein in most *E. coli* strains (1), and our attempts failed to knockout *nusG* in several *E. coli* strains. Thus, we instead developed a NusG depletion strategy by repressing transcription of *nusG* in its original locus with an inducible P_{ara}-dCas9 protein roadblock guided to the translation start codon of *nusG* by *nusG*-sgRNA (2). Induction of the dCas9 roadblock with arabinose resulted in rapid reduction in *nusG* transcription and time-dependent depletion of NusG protein with almost complete loss within 4 hours when the cells ceased to grow (*SI Appendix,* Fig. S1). Even without arabinose-dependent induction of dCas9, the NusG protein level in our depletion strain was lower than the isogenic WT strain that lacked the *nusG*-targeting sgRNA, indicating incomplete repression of the dCas9/ *nusG*-sgRNA system in the absence of arabinose (*SI Appendix,* Fig. S1*A*). Therefore, we used RNET-seq data generated from a strain lacking the *nusG*-sgRNA as a surrogate for a WT control. We generated RNET-seq data from three biological replicates of WT and NusG-depleted (dNusG) cells.

Bioinformatic pipelines for analysis of RNET-seq data. All applications for RNET-seq were deployed on the DNANexus platform (3). The Aligner 2.1 pipeline was used to align sequence reads to the reference NC_000913.2 *E. coli* genome and to remove reads that mapped to more than a single genomic location. The Aligner 4.1 pipeline was used to align all sequence reads to the genome including reads that mapped to multiple genomic locations such as rRNA genes. Finder 2.2 was used to identify pause sites according to minimum threshold values for pause score and count (3). The Differential_Pauses pipeline was developed in this work for analyzing differential pause site strength. Following our previously developed bioinformatic definition of pause peaks in RNET-seq data, we calculated the pause score values for each position in the genome as a ratio of the count of a unique 3' end to the median count in the 100 bp region centered at the 3' end (3). We considered an RNET-seq peak possessing a score above a threshold value as an authentic transcription pause site. The minimum read counts for pauses that qualified as pause sites were automatically calculated as 597 for data derived from WT cells and 533 for data derived from dNusG cells and the minimum score value was set to 50. This stringent cutoff for genome-wide analysis resulted in a total of 1613 pauses in WT cells and 5091 pauses in dNusG cells (SI Appendix, Dataset S1). Adjacent pauses within a 10 nt window may be considered as a single pause site to account for 1-5-nt heterogeneity in position of the 3' RNA ends observed at some pause sites (SI Appendix, Dataset S2).

All instances of false unique pauses derived from single-nucleotide polymorphisms in rRNA and tRNA genes were removed manually from the Finder-generated lists of strong pauses (score values ≥50). Next, we categorized the strong pauses as unique to WT, dNusG, and shared by both WT and dNusG cells. We identified 4408 pauses unique to dNusG cells and 444 unique to WT cells (SI Appendix, Dataset S3). Among the 444 pauses unique to WT cells, 361 had a single 3' end and 83 had multiple 3' ends clustered within a 10-bp window. According to our definition, such clustered pauses likely derived from the same local sequence elements in the DNA and nascent RNA and we further discuss their origin in SI Appendix, Fig. S18 (3). We also identified 509 pauses that were shared by WT and dNusG cells (SI Appendix, Dataset S3). Only a fraction of these pauses was affected by NusG depletion.

Setting minimum values for pause score and read count to 12 (including weak pause sites) and 200 (including less abundant transcripts), respectively, resulted in a total of 17243 pauses in WT cells and 27449 in dNusG cells (SI Appendix, Dataset S4). This less stringent cutoff identifying weaker pauses was used to populate short genomic regions such as 5' UTRs and translation initiation regions with pauses, which otherwise had an insufficient number of pauses for reliable data analyses.

To determine how NusG depletion affected the pause strength, we generated differential pause tables comparing the pause scores at each pause in WT and dNusG cells. As the majority of the pauses were unique to either WT or dNusG cells, the impact of NusG depletion was determined as a ratio of score values in dNusG versus WT cells even if the pause at the corresponding genomic position was observed only in one type of cell. The stringent cutoff of score values ≥50 resulted in 4301 differential pauses (SI Appendix, Dataset S5). The relaxed cutoff of score values ≥12 resulted in 28171 differential pauses (*SI Appendix*, Dataset S6). A

summary of information from Datasets 1 to 6 is presented in SI Appendix, Table S1. The strength of the *hisL* pause site, which has been extensively characterized *in vitro* (4), follows the time course of NusG depletion (*SI Appendix*, Fig. S17).

In general, the Differential_Pauses pipeline indicated that pauses unique to WT cells corresponded to NusG-stimulated pauses and pauses unique to dNusG cells corresponded to NusG-suppressed pauses (*SI Appendix*, Datasets S3 and S5). Data generated by Differential_Pauses generally agreed with the data generated by Finder.

NusG suppresses pausing in rRNA operons. NusG is a component of a multiprotein transcription antitermination complex that forms during transcription of seven rRNA operons in *E. coli,* making it impossible to assign RNET-seq reads to a specific rRNA operon (5). However, RNET-seq read coverage of all seven rRNA operons was reduced 2- to 3-fold (2.7-fold average) in dNusG cells, consistent with a role of NusG in antitermination (*SI Appendix*, Fig. S15*A*). NusG depletion also increased the strength of several pauses in the rRNA operons, but only the single strongest pause in dNusG cells passed the stringent cutoff score value of ≥50. This pause, which was not observed in WT cells, was located at G1025 in a bulge between helix 41 and helix 42 within domain II of mature 23S rRNA (6). Except for possessing a G residue at the 3' end, the sequence of the G1025 pause was similar to the -10G/-9G…-1Y +1G/T logo of NusG-suppressed pauses from protein-coding regions (*SI Appendix*, Fig. S15*B*). Helix 41 of 23S rRNA is formed by base paring between nts 991-1018 with nts 1144-1163 (*SI Appendix,* Fig. S15*C*). We conclude that the overall effect of NusG depletion on pausing in rRNA operons was moderate, compared to the effect on pausing within protein-coding genes.

Impact of transcript cleavage factors GreA and GreB on pausing *in vivo*. *E. coli* Gre factors rescue backtracked transcription complexes by stimulating the intrinsic endonucleolytic cleavage activity of RNAP such that the newly formed 3' end of the nascent transcript is properly aligned in the catalytic center of RNAP (7). We reasoned that the effect of NusG on backtracked pauses could be underestimated *in vivo* due to the robust rescue of these pauses by Gre factors, as was shown previously by RNET-seq comparing WT and ∆*greA* ∆*greB* strains (8, 9). This concern was derived from sequence bias that we observed at position +1 in the sequence logo of pause sites (Fig. 3*C-D*). The +1T residue was almost equally frequent in the logo as the reported +1G, but +1T was not a part the previously identified consensus pause motif -10G -1Y +1G. This potential bias reinforced our concern about the impact of 3' RNA end processing by Gre factors at these sites (*SI Appendix*, Table S1) (8, 10-11). To further investigate the possible impact of Gre factors, we generated separate sequence logos of NusG-suppressed pauses containing +1A, +1C, +1G or +1T. This position within the transcriptional bubble is located adjacent to the downstream edge of the RNA-DNA hybrid in the transcription elongation complex. This analysis revealed that a +1 pyrimidine (T or C) correlated with the presence of the -9G residue in the logo, whereas a +1 purine (G or A) correlated with the presence of -10G and -1 pyrimidine residues in the logo (*SI Appendix*, Fig. S10*A-D*). Note that -10G and -9G are at the upstream edge of the RNA-DNA hybrid in RNAP (Fig. 4*B-D*). The pauses with -9G +1T residues likely corresponded to backtracked elongation complexes that were subsequently cleaved upstream of a U residue in RNA by Gre factors. This hypothesis was confirmed by analyzing sequence logos of pauses enriched in different read lengths in our libraries. Backtracked pauses result in RNET-seq read lengths of >18 nt (3, 8). Gre factor-stimulated trimming of nascent RNA at the 3' end *in vivo* or in crude cell lysates of WT and dNusG cells was expected to reduce the read length. The finding that >18 nt reads (backtracked but not processed by Gre factors) were enriched with -10G, -1Y, and +1G sequences is consistent with our hypothesis. In contrast, 17 nt reads that were likely products of the 3' RNA cleavage, were enriched in -9G and +1T residues (Fig. 4*B-D*). A similar but less pronounced tendency was observed for >18 nt and 17 nt reads of NusG-suppressed pauses (*SI Appendix*, Fig. S10*E-F*). The observed patterns strongly suggest that reads >18 nt belonged to backtracked complexes, and 17 nt reads, at least in part, were the products of preferential Gre factor-mediated cleavage 5' of the U residue.

Among pairs of two adjacent pauses separated by 1-2 nts, the downstream pauses generally possessed a larger fraction of backtracked (>18 nt) reads and a smaller fraction of 17 nt reads compared to the upstream pauses (*SI Appendix*, Table S6). Comparison of 3' ends of

several selected NusG-suppressed pauses in the WT strain (this work) and the *greA greB* knockout strain (9) confirmed that pausing at the -1Y +1G sequence context of the consensus motif remained intact in Δ*greA* Δ*greB* cells, whereas subsequent Gre factor-stimulated cleavage in WT cells enriched the elongation complex register with +1T (*SI Appendix*, Fig. S18). *In vitro* transcription confirmed that pausing occurred at the position observed *in vivo* for several pause sites that we tested (*SI Appendix*, Table S7). For some pauses, *in vitro* transcription reveled pausing 1 to 4 nt downstream of the position detected *in vivo*, providing compelling evidence of the involvement of Gre factors at these sites *in vivo*. In these cases, the sequence of *in vitro* pauses fits well with the consensus motif -10G -1Y +1P, consistent with this position being the initial pause followed by backtracking and cleavage *in vivo* (*SI Appendix*, Table S7). Based on the occurrence of +1T and +1G residues in the sequence logos, Gre factors impacted NusG-suppressed and NusG-independent pause sites almost equally (Fig. 3*C-D*). Note that backtracked complexes of RNAP are the target of Gre factors (7). Finally, our data showed no correlation between the influence of NusG on pause strength and backtracking (*SI Appendix*, Fig. S14). Collectively, Gre-stimulated cleavage of nascent transcripts participated in the generation of nascent RNA 3' ends at some pause sites but did not overshadow the general suppression of pausing by NusG *in vivo*. Further validation of this hypothesis would require engineering of the dNusG *E. coli* strain lacking the *greA* and *greB* genes, which is beyond the scope of this study.

Materials and Methods

Strains, plasmids, and oligonucleotides. *E. coli* strains used in this study are listed in *SI Appendix*, Table S8. Plasmids used in this study are described in *SI Appendix*, Table S9. Sequences of DNA and RNA oligonucleotides used in this study are described in *SI Appendix*, Table S10.

Construction of the *nusG* knockdown strain. *E. coli* train SJ_XTL219 is a tCRISPRi derivative of MG1655 for blocking expression (knockdown) of chromosomal genes by induction of a chromosomally integrated *dcas9*, which encodes endonuclease-deficient Cas9, and recombineering of *sgRNA*. *dcas9* was inserted into the *ara* operon under control of the arabinose-inducible P_{BAD} promoter. Linked to a *tet-sacB* counter-selectable marker, the sgRNA sequence contained the Cas9-binding and transcription terminator modules that were constitutively expressed from the *galM* region of the chromosome (2).

rpoC carrying a 3'-terminal 6His-tag sequence was introduced from strain NB854 (8) into strain SJ_XTL219 using P1 transduction and selection for a linked Km^r resistance marker. The resulting strain (NB1246) was used as the NusG depletion strain for RNET-seq.

The PAM-following sequence AAAAAGCGCTGGTACGTCGT in *nusG* was selected for targeting by dCas9. This sequence was embedded into the 90-mer targeting oligonucleotide sgRNA-*nusG* adjacent to the dCas9 handle and flanked by 35-nt chromosome homology regions (*SI Appendix*, Table S10). sgRNA-*nusG* was created by replacement of the *tet-sacB* cassette in strain NB1246 with the targeting oligonucleotide by recombineering using the lambda RED functions from plasmid pSIM18 (15, 16). Colonies were selected on LB agar plates without NaCl but containing 6% sucrose and additionally screened for Tet-sensitivity. Replacement of the *tet-sacB* cassette with the *nusG*-specific sgRNA was confirmed by PCR. Removal of plasmid pSIM18 by several passages of cells on LB plates at 37°C was confirmed by hygromycin sensitivity on LB plates supplemented with 200 µg/ml hygromycin. Induction of *dcas9* in the resulting strain (NB1247) with arabinose results in a transcriptional roadblock that represses transcription of *nusG*.

DNA templates and proteins used for *in vitro* transcription. Selected pause sites for analysis by *in vitro* transcription were fused with a strong engineered promoter followed by a C-less cassette. The resulting constructs were tested for pausing by single-round *in vitro* transcription. Templates for *in vitro* transcription were generated via PCR by merging two overlapping fragments as described previously (3). The first fragment containing the pause site of interest surrounded by its flanking regions was amplified using *E. coli* chromosomal DNA as the template and a pair of site-specific primers. The second fragment contained a consensus promoter with an

extended -10 region, followed by a 29 nt C-less cassette and a sequence that forms a strong RNA hairpin when transcribed, which insulated the promoter-proximal region of the transcript from the downstream sequence. The promoter/insulator sequence was a derivative of the *B. subtilis trp* leader amplified from plasmid pAY196 and common to all tested pause sites (17, 18). PCR fragments were fractionated on precast 8% TBE polyacrylamide gels for 45 min at 200 V. Gels were stained with SyBR Gold (Life technologies) and bands of the correct length were excised from the gel. DNA was extracted with 60 µl of buffer containing 3 mM Tris-HCl, pH 8.9, 0.3 mM EDTA and 50 mM betaine by shaking at 1250 rpm for 1 hr at 60°C. Ten µl of the pause site-containing fragment and 1.2 µl of the promoter-containing fragment were combined in a 48 µl PCR reaction to merge the overlapping fragments using the promoter forward primer and the pause site reverse primer. The resulting DNA templates were recovered using the QIAquick PCR purification kit (Qiagen) and eluted with 50 µl of 0.5x TE buffer. *E. coli* RNAP holoenzyme was purified from strain NB959 carrying a 3'-terminal 6His-tag sequence at the *rpoC* gene (8). Previously described recombinant NusG protein (19) was overproduced and purified by the Protein Purification Core (Frederick National Lab Laboratory Services).

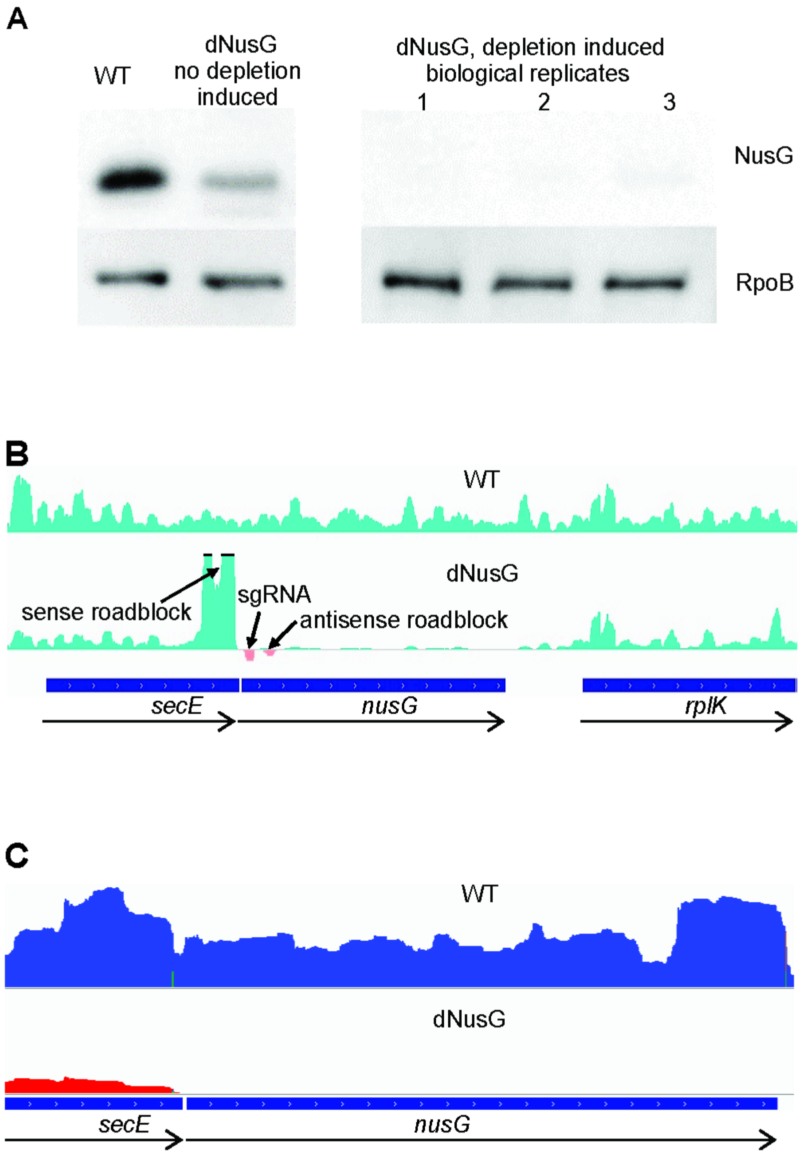

Fig. S1. NusG depletion after dCas9 induction with arabinose. (*A)* Top panel, Western blot analysis indicates that the level of NusG was substantially higher in the WT strain compared with the depletion strain without dCas9 induction, indicating partial leakage of the P_{BAD} promoter in the absence of arabinose induction. Four hours of dCas9 induction with arabinose resulted in NusG depletion below detectable levels in all samples used for RNET-seq. The image displays 3 biological replicates for the induced strain after probing for NusG. The WT parental strain was used as a control. Bottom panel, Western blot for the β subunit of RNAP (RpoB) as a loading control. *(B)* RNET-seq read coverage displayed in IGV browser showed a dramatic inhibition of transcription of *nusG* upon dCas9 induction. RNET-seq detected the *nusG*-targeting sgRNA (antisense to the coding sequence), as well as strong pausing of RNAP near the translation start codon of *nusG* and immediately upstream from the dCas9 roadblock. *(C)* RNA-seq data displayed in IGV showed the complete absence of the full-length *nusG* mRNA and a substantial repression of the upstream *secE* mRNA upon NusG depletion. The latter phenomenon might be derived from delayed release of the *secE* transcript from dCas9-blocked RNAP and reduced

stability of the corresponding mRNA lacking the stabilizing RNA secondary structure near its 3' end.

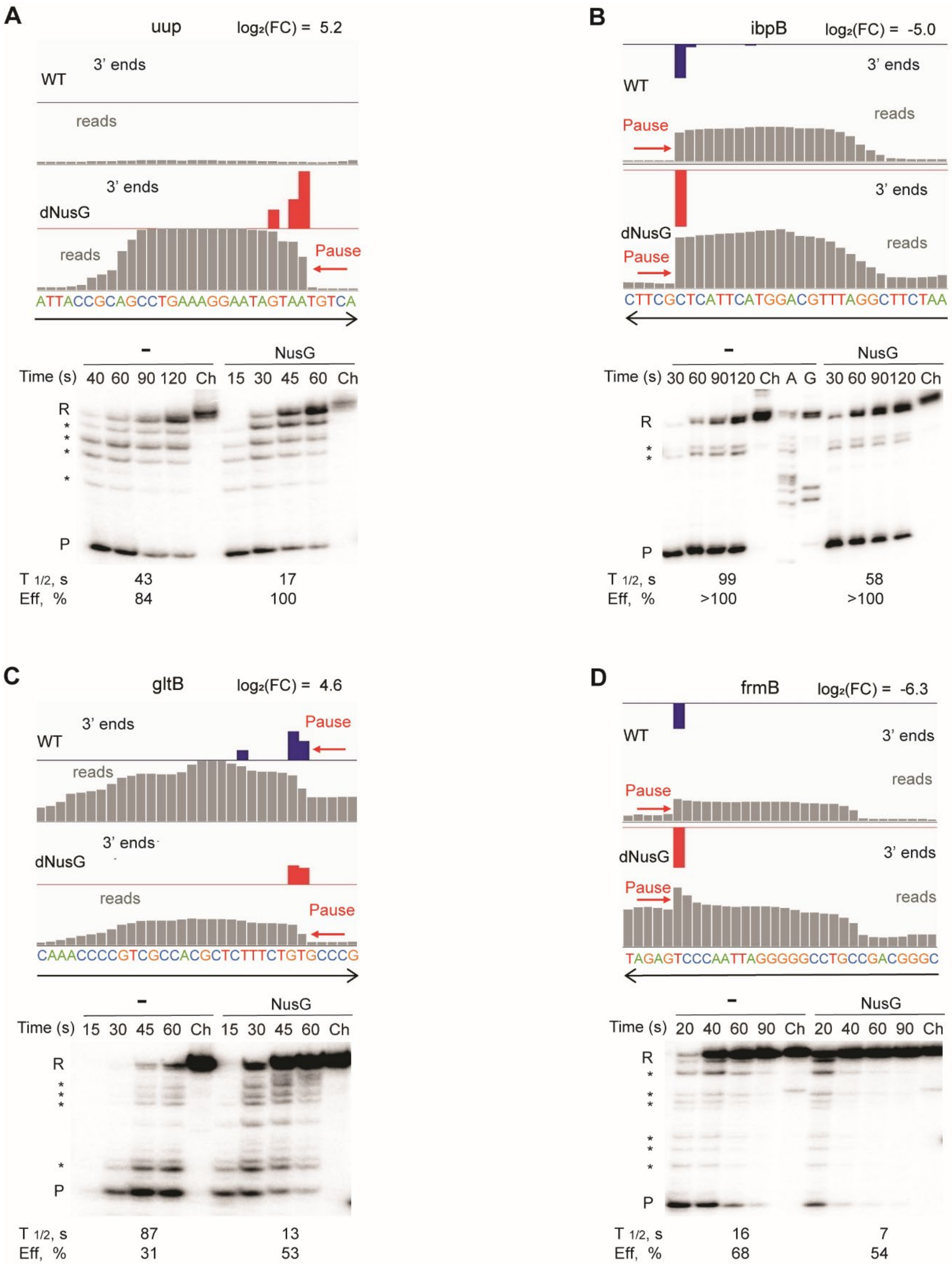
A
uup
log₂(FC) = 5.2
3' ends
WT
reads
3' ends
dNusG
reads
Pause
ATTACCGCAGCCTGAAAGGAATAGTAATGTCA
-
NusG
Time (s) 40 60 90 120 Ch 15 30 45 60 Ch
R
P
T 1/2, s 43 17
Eff, % 84 100
B
ibpB
log₂(FC) = -5.0
3' ends
WT
reads
Pause
3' ends
dNusG
reads
Pause
CTTCGCTCATTCATGGACGTTTAGGCTTCTAA
-
NusG
Time (s) 30 60 90120 Ch A G 30 60 90120 Ch
R
P
T 1/2, s 99 58
Eff, % >100 >100
C
gltB
log₂(FC) = 4.6
3' ends
WT
Pause
reads
3' ends
dNusG
reads
Pause
CAAACCCCGTCGCCACGCTCTTTCTGTGCCCG
-
NusG
Time (s) 15 30 45 60 Ch 15 30 45 60 Ch
R
P
T 1/2, s 87 13
Eff, % 31 53
D
frmB
log₂(FC) = -6.3
WT
3' ends
reads
Pause
dNusG
3' ends
Pause
reads
TAGAGTCCCAATTAGGGGGCCTGCCGACGGGC
-
NusG
Time (s) 20 40 60 90 Ch 20 40 60 90 Ch
R
P
T 1/2, s 16 7
Eff, % 68 54

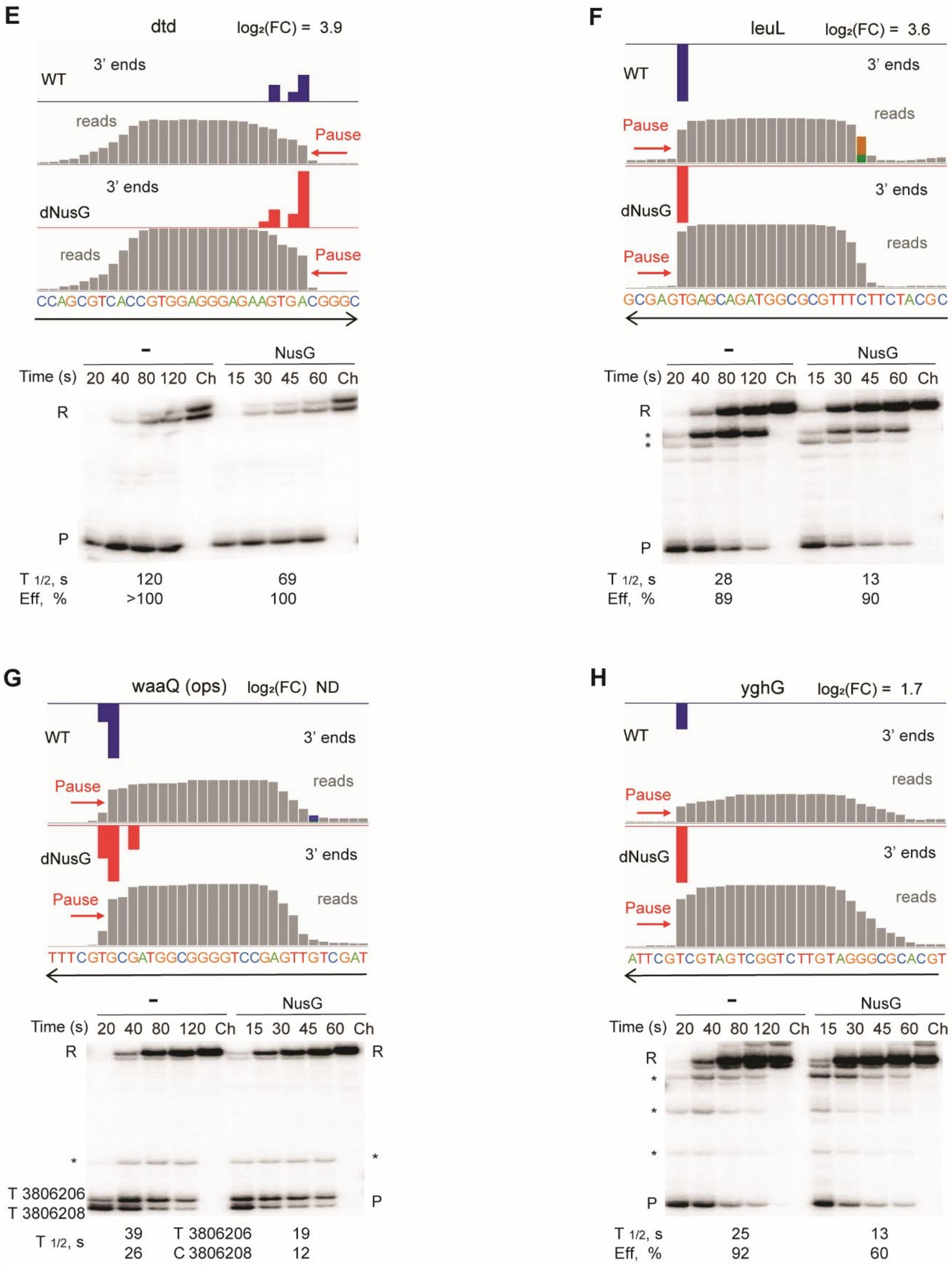

Fig. S2. ***In vitro*** **validation of NusG-suppressed and NusG-stimulated pause sites.** Pause sites in the *uup (A)*, *ibpB (B)*, *gltB (C)*, *frmB (D,) dtd (E)*, *leuL (F)*, *waaQ (ops) (G)*, and *yghG (H)* genes in WT and dNusG cells identified by RNET-seq *in vivo* as they appear in the IGV browser (top of each panel). Black arrows indicate the direction of transcription. Genome-aligned reads are in gray while mapped 3′ ends corresponding to the RNAP active site are in blue and red for

WT and dNusG data, respectively. Log_2 values of fold-change in the pause score upon NusG depletion (log_2FC) are indicated except for the *waaQ (ops)* site that was not detected by our Differential Pauses pipeline (ND). Values are positive for NusG-suppressed sites and negative for NusG-stimulated sites. Sequence around the pause sites is indicated. The bottom of each panel shows the results of a single-round *in vitro* transcription pause assay of the indicated templates in the absence and the presence of NusG (±NusG). Time points of the reaction are indicated above each lane. Ch, chase reactions. P, pause band; R, run-off transcript; *, minor pauses. The pause half-life (T ½) and efficiency (Eff) are indicated below each set of lanes. Efficiency exceeding 100 % is a consequence of data fitting. Pausing at two residues (T 3806206 and C 3806208 in the *E. coli* reference genome NC_000913.2) are observed *in vitro* at the *waaQ* (*ops*) pause site (20), the pause half-lives are indicated for both pauses.

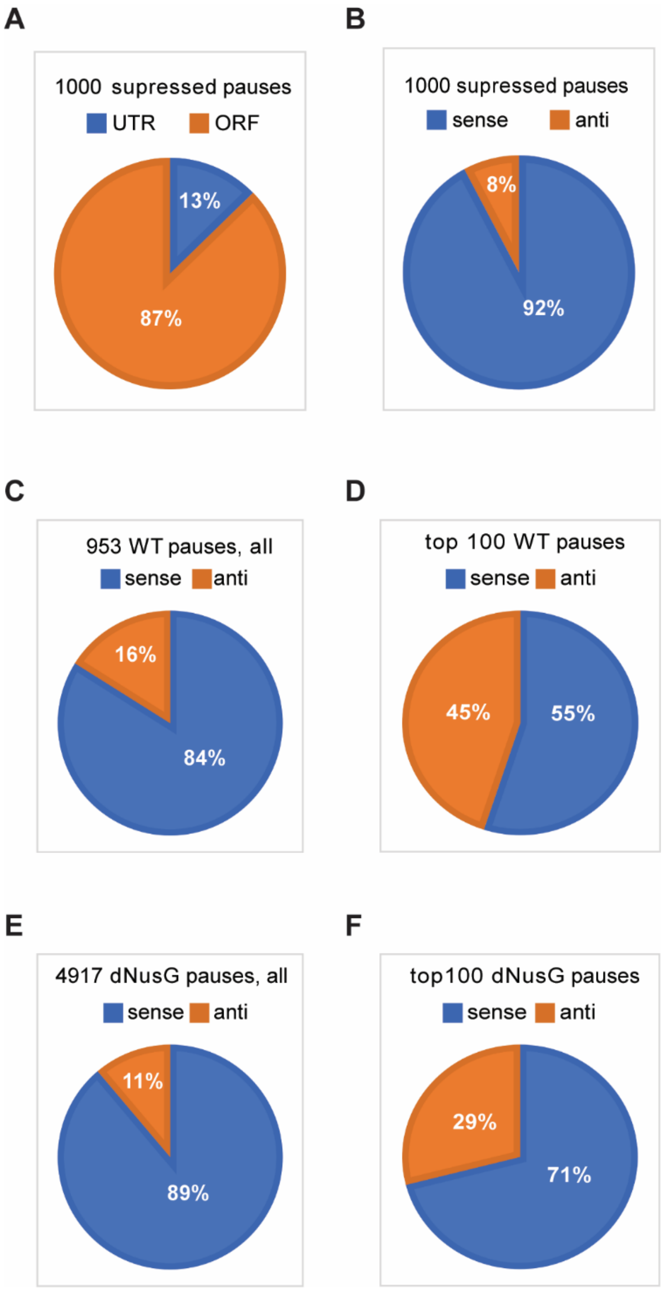

Fig. S3. Distribution of strong pauses (score >50) among untranslated (UTR), protein-coding (ORF), sense, and antisense transcription units. *(A)* Distribution of NusG-suppressed pauses among UTRs and ORFs (*SI Appendix*, Dataset S5). *(B)* Distribution of NusG-suppressed pauses among sense and antisense transcription units (*SI Appendix*, Dataset S5). Distribution of all pauses *(C)* and the top 100 pauses with the highest score *(D)* from WT cells among sense and antisense transcription units (*SI Appendix*, Dataset S3). Distribution of all pauses *(E)* and the top 100 pauses with the highest score *(F)* from dNusG cells among sense and antisense transcription units (*SI Appendix*, Dataset S3).

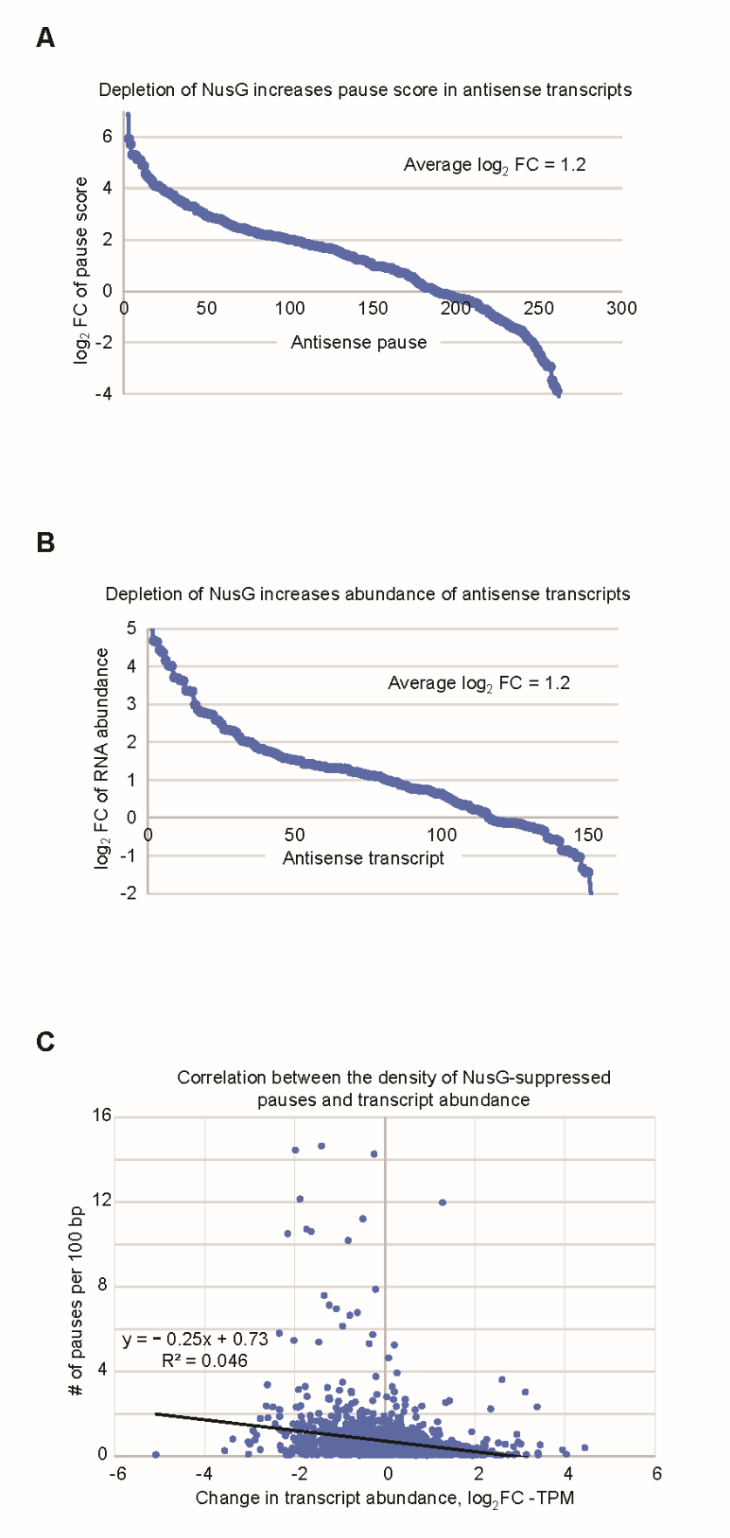

Fig. S4. NusG affects antisense transcription and the abundance of nascent RNA. *(A)* Changes in the pause score of 266 antisense pauses upon NusG depletion are plotted in decreasing order. NusG depletion generally makes pauses stronger and results in an average increase in the pause score ~2-fold ($log_2FC=1.2$). However, a few pauses decrease in strength

and have log_2FC values below 0. Data are from column V (log2FC-Pause-TPM) of *SI Appendix*, Dataset S5. *(B)* Changes in the abundance of 151 antisense transcripts upon NusG depletion are plotted in decreasing order. NusG depletion generally stimulates antisense transcription and results in an average increase in the abundance of antisense RNA ~2-fold ($log_2FC=1.2$). However, a few pauses decrease in abundance and have log_2FC values below 0. Data are from the column J (Log2FC-CDS TPM) of *SI Appendix*, Dataset S5. *(C)* Negative correlation between the density of NusG-suppressed pauses and transcript abundance upon NusG depletion. Values of changes in transcript abundance are from RNET-seq data in the column J (Log2FC-CDS TPM) of *SI Appendix*, Dataset S5.

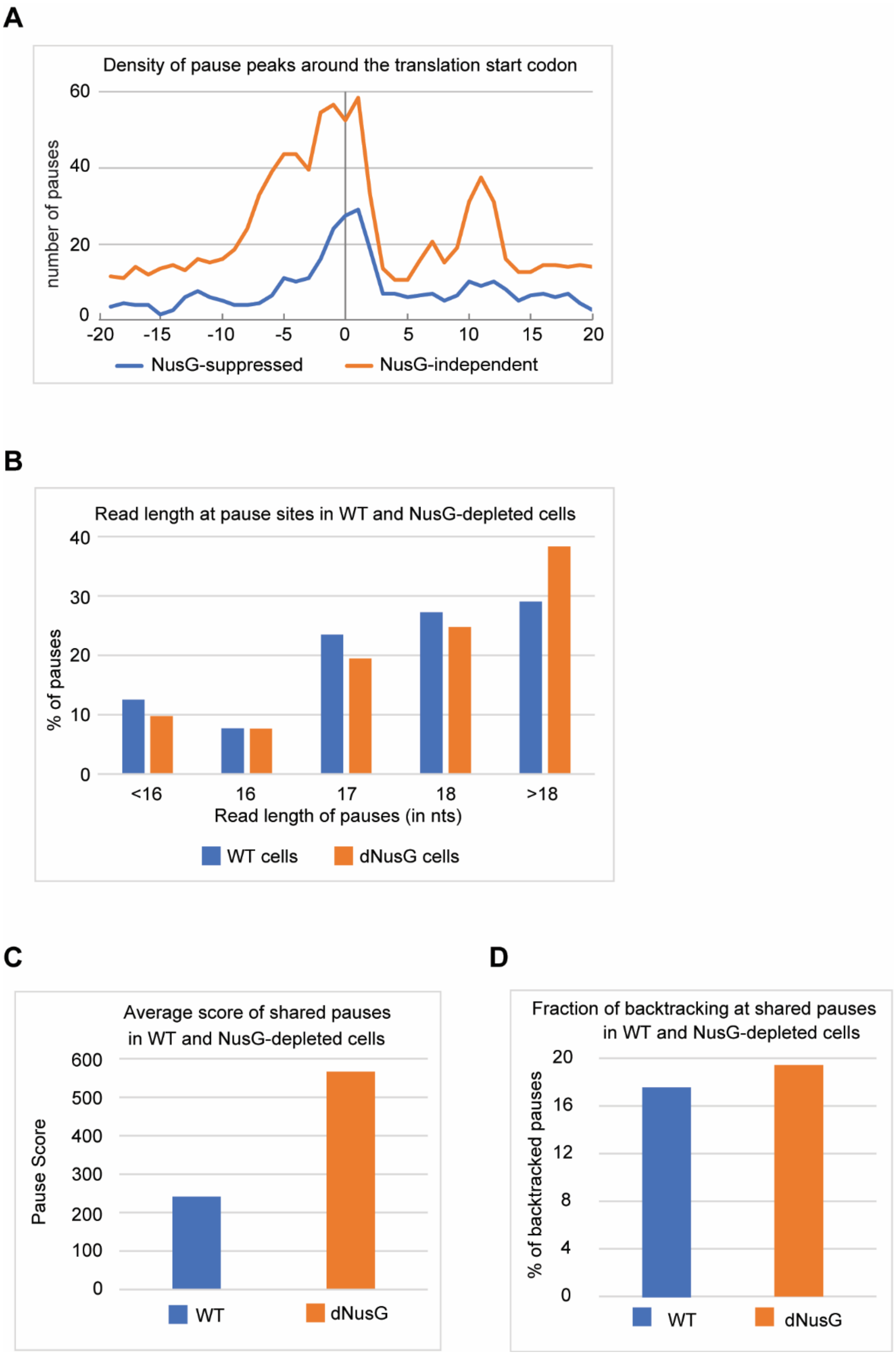

Fig. S5. Effects of NusG depletion on genome-wide pausing and backtracking of RNAP. *(A)* Density of NusG-suppressed and NusG-independent pauses around the translation start codon (from *SI Appendix*, Dataset S6). Position "0" corresponds to the first residue of the start codons. Experimentally derived values are plotted using a two-period moving average. *(B)* Read length

distribution at pauses in WT and dNusG cells (from *SI Appendix*, Dataset S3). Longer than 18 nt reads are assigned to backtracked elongation complexes. Average pause score *(C)* and fraction of backtracked pauses *(D)* of pauses shared by WT and dNusG cells (from *SI Appendix*, Dataset S3). Pauses are considered as backtracked if >50% of all reads derived from the pause site are longer than 18 nucleotides.

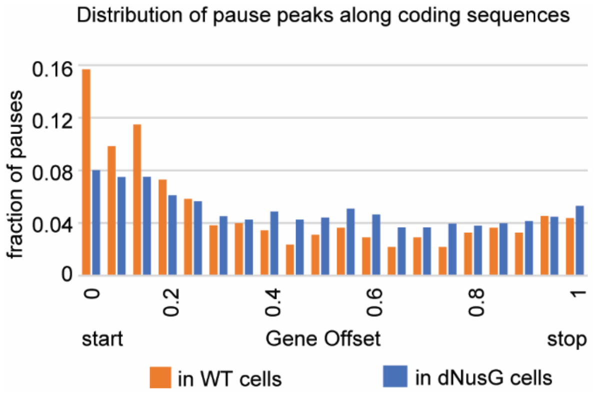

Fig. S6. Distribution of pauses along ORFs. Pauses in WT cells are enriched at the beginning of ORFs, whereas pauses in dNusG cells are more evenly distributed along ORFs.

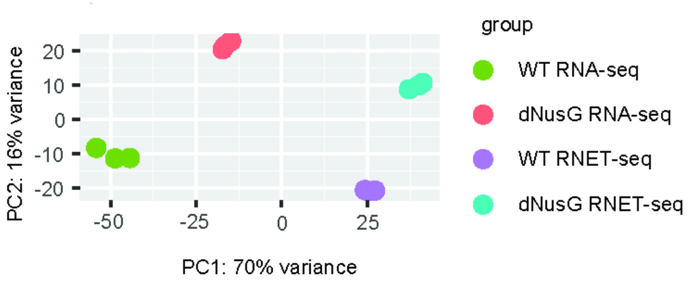

Fig. S7. Principal component analysis (PCA) of RNET-seq and RNA-seq data for differential gene expression in WT and dNusG cells. Three biological replicates of RNET-seq and four biological replicates of RNA-seq form 4 separate PCA clusters. 70% variance in PC1 reflects the difference between RNA-seq and RNET-seq data, whereas the 16% variance in PC2 reflects the difference between WT and dNusG cells. PCA was performed using RNET-seq and RNA-seq data from *SI Appendix*, Dataset S7.

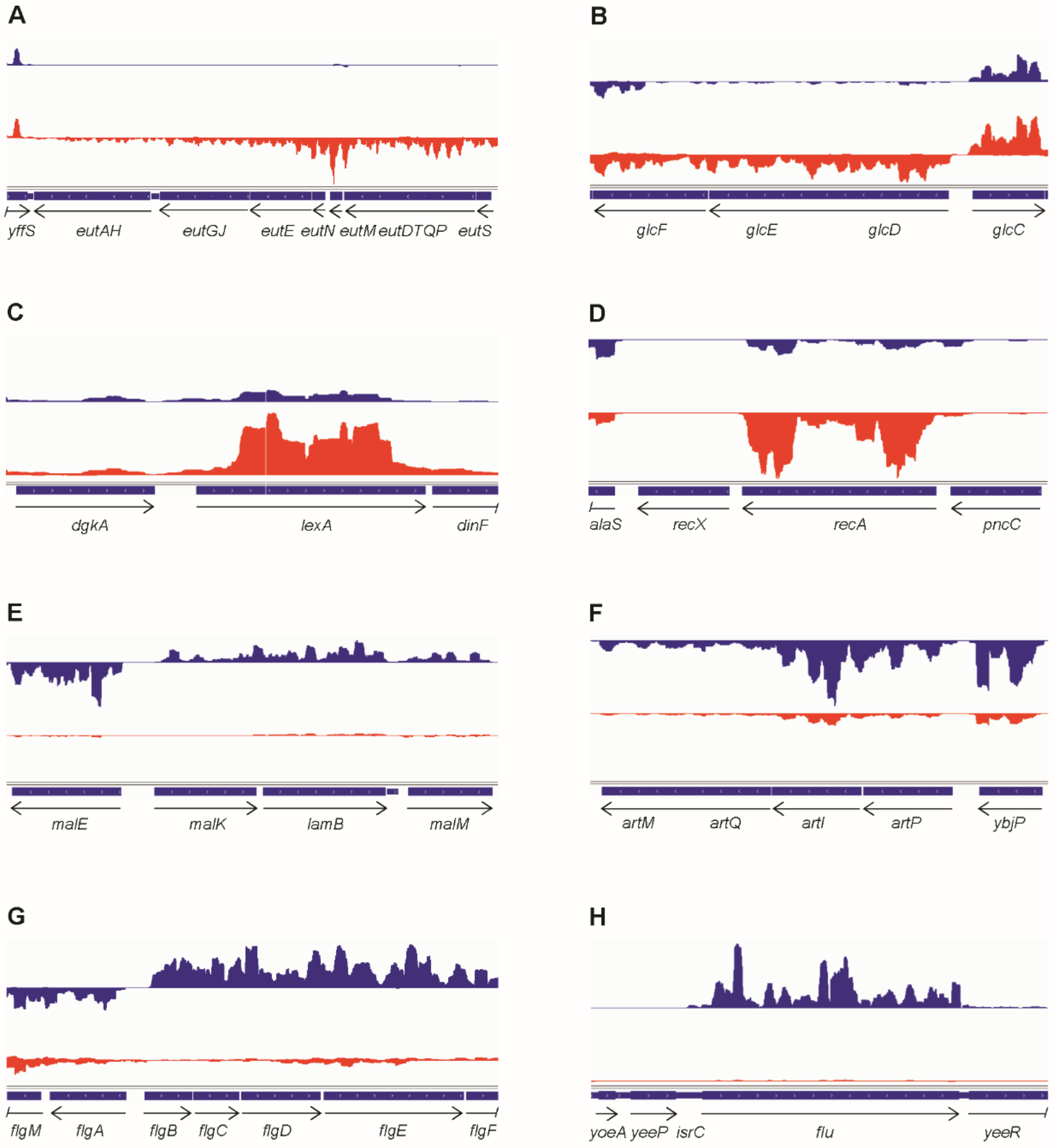

Fig. S8. NusG depletion affects expression of numerous genes. Affected genes were identified by RNA-seq using differential gene expression and Gene Ontology analysis (biological process in *Escherichia coli* at http://geneontology.org/). The RNA-seq read coverage data of WT cells (blue) and dNusG cells (red) as they appear in the IGV browser. Expression of genes for primary alcohol catabolism such as ethanolamine metabolic process *(A)* and glycolate metabolism *(B)*, as well as some genes involved in the SOS response to DNA damage such as *lexA (C)* and *recA (D)* were increased after NusG depletion. Conversely, expression of genes involved in maltodextrin/maltose transport *(E)*, arginine transmembrane transport *(F)*, flagellum-dependent motility *(G)*, and aggregation/biofilm formation *(H)* were decreased after NusG depletion.

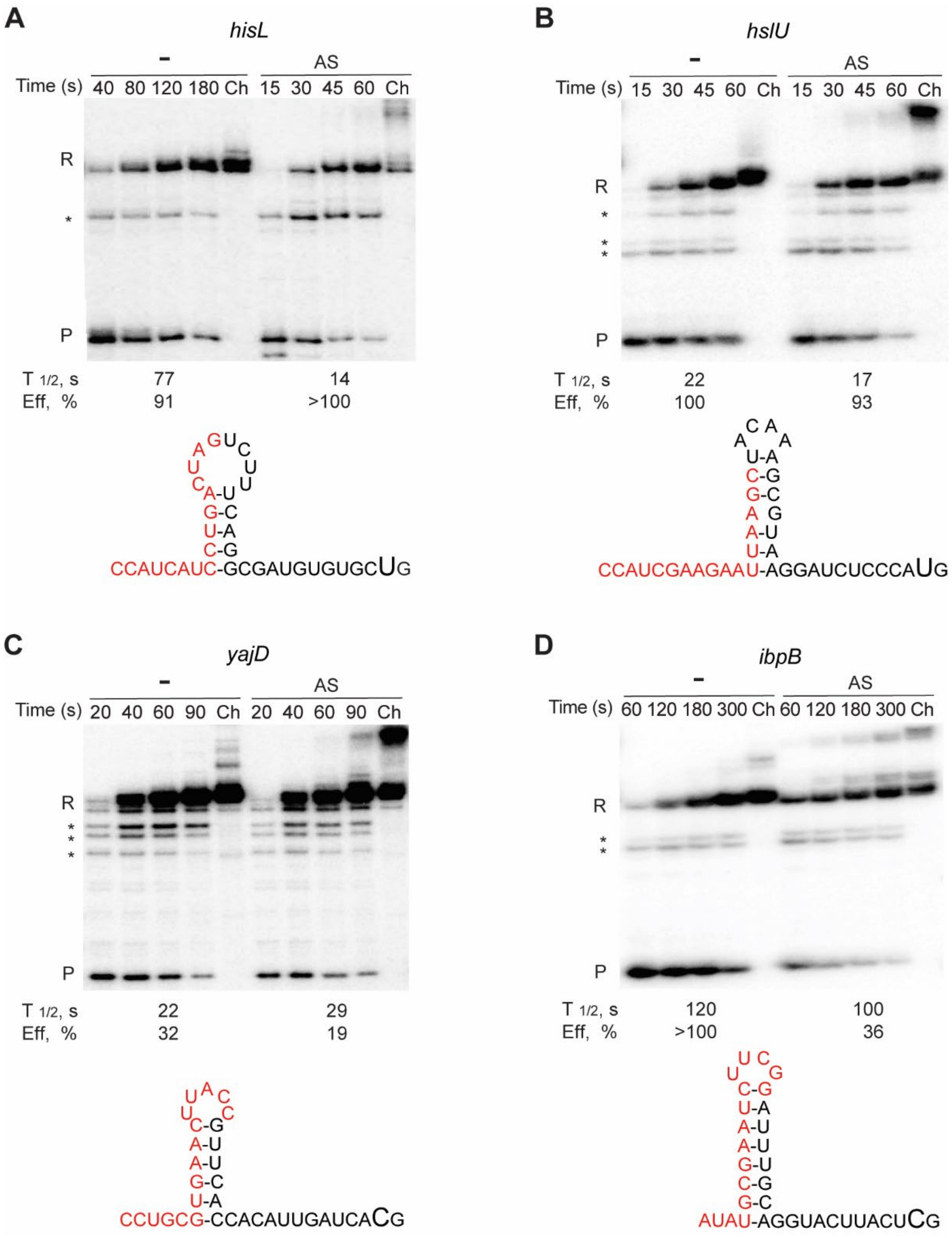

Fig. S9. RNA hairpins that fold upstream of pause sites stimulate pausing. Time points of single-round *in vitro* transcription assays are indicated above each lane. P, pause band; R, run-off transcript; *, minor pauses. The pause half-life ($T_{1/2}$) and efficiency (Eff) are shown below each set of lanes. Antisense oligonucleotides designed to inhibit formation of a predicted pause hairpin upstream of the pause site decreased the pause half-life and/or efficiency. Transcription was performed using the indicated templates in the absence (–) or presence (AS) of an antisense oligonucleotide. Template switching by RNAP is probably responsible for transcription products in the presence of an antisense oligonucleotide that are longer than the R transcript (3). The bottom

of each panel shows the predicted pause hairpins. The residues that are complementary to the AS oligo are in red and the pause 3' ends are in larger font.

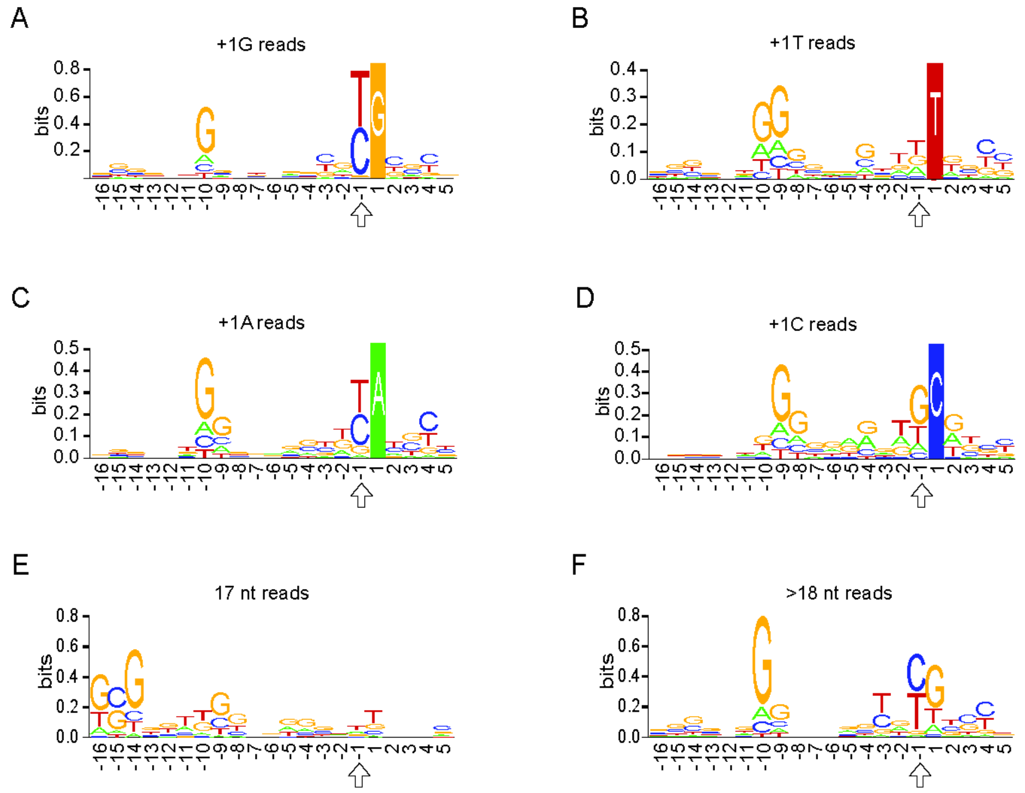

Fig. S10. Sequence logo analysis of various classes of NusG-suppressed pauses. Sequence logos were generated separately using pauses coding for G *(A),* T *(B)*, A *(C)*, or C *(D)* residue at the +1 position. Logo of the NusG-suppressed pauses containing 17 nt reads *(E)* and >18 nt reads *(F)* are also shown. Position -1 corresponds to the 3' end of paused RNA and is marked with an arrow.

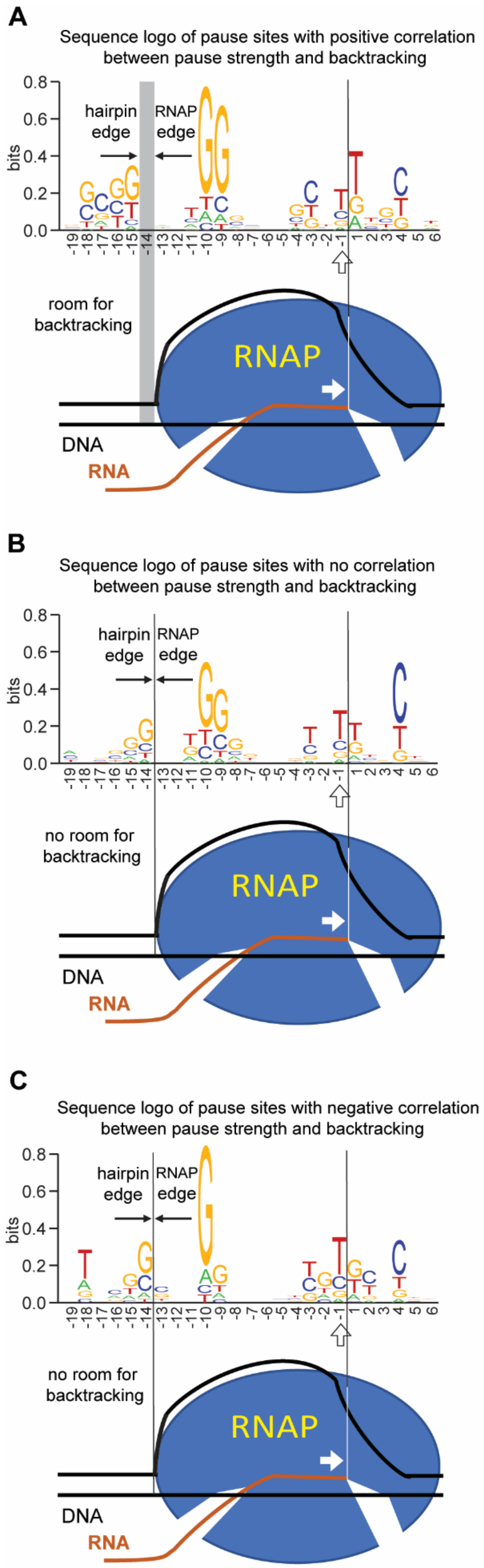
A
Sequence logo of pause sites with positive correlation between pause strength and backtracking
bits
hairpin edge
RNAP edge
room for backtracking
RNAP
DNA
RNA
B
Sequence logo of pause sites with no correlation between pause strength and backtracking
bits
hairpin edge
RNAP edge
no room for backtracking
RNAP
DNA
RNA
C
Sequence logo of pause sites with negative correlation between pause strength and backtracking
bits
hairpin edge
RNAP edge
no room for backtracking
RNAP
DNA
RNA

Fig. S11. The hairpin to 3' end distance of pauses affects backtracking and the effect of NusG. Three groups of 50 NusG-suppressed pause sites with a positive (*A*), negligible (*B*), or negative (*C*) correlation between NusG-suppressed pausing and backtracking, respectively, are listed in *SI Appendix*, Dataset S8. *(A)* NusG depletion increased the fraction of backtracked reads at sites where the hairpin to 3' end distance was 14 nt. *(B, C)* Pauses with a hairpin to 3' end distance of 13 nt inhibits backtracking such that the fraction of backtracked reads after NusG depletion remained unchanged.

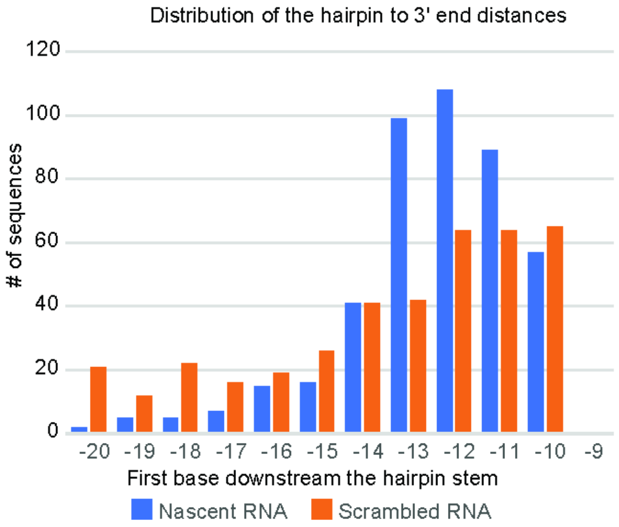

Fig. S12. Distribution of the hairpin to 3' end distances at NusG-suppressed pauses. 458 NusG-suppressed pauses that possess dNusG Score values above 300 and log_2FC-Pause-TPM values above 3 were selected from *SI Appendix*, Dataset S5. These sequences were scrambled to make a negative control. Prediction of RNA folding was performed using CLC Genomics Workbench software (Qiagen, https://resources.qiagenbioinformatics.com/manuals/clcgenomicsworkbench/current/index.php?manual=Annotate_with_Flanking_Sequence.html). The structure closest to the 3’ end was selected if several alternative hairpins were predicted for a single pause site.

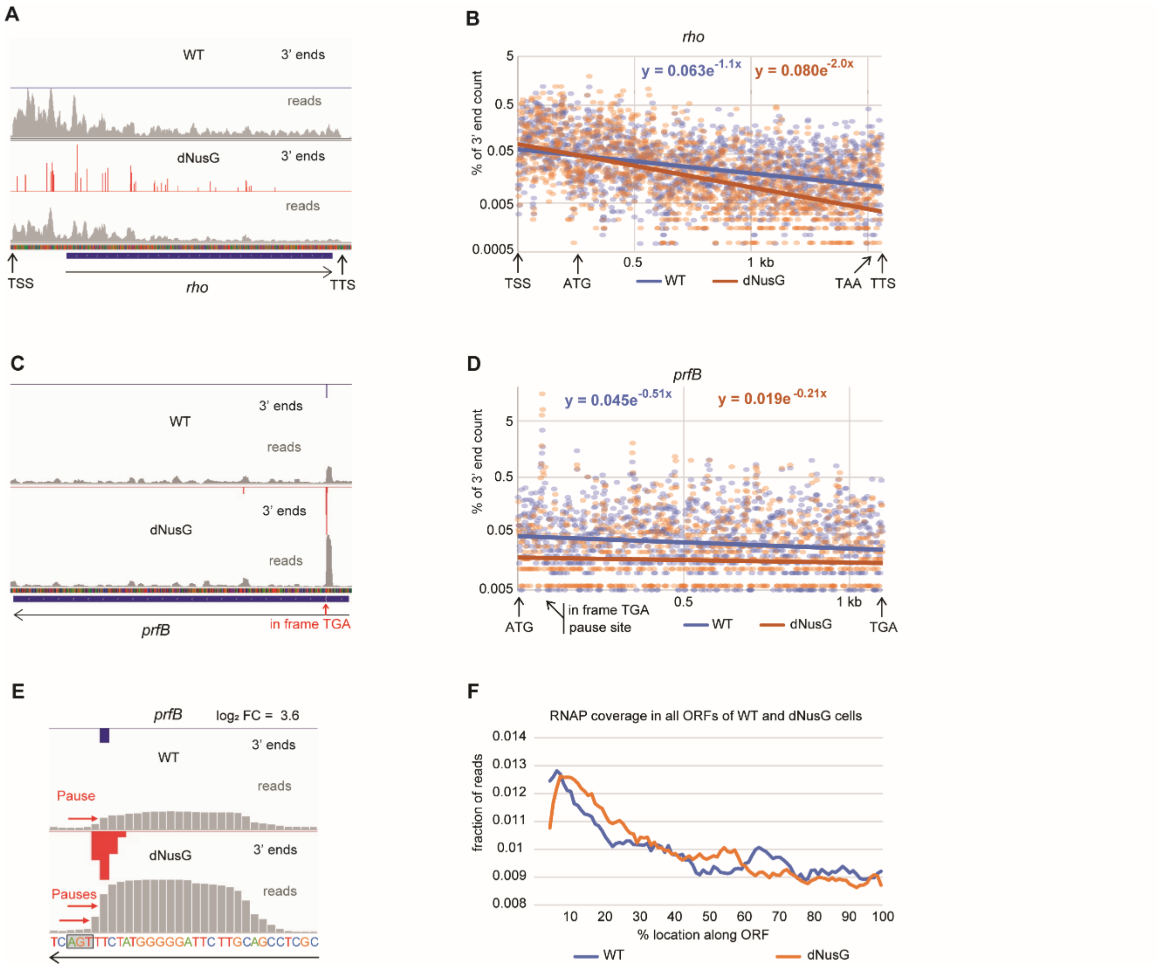

Fig. S13. NusG depletion exhibits opposite effects on transcriptional polarity of *rho* and *prfB*. Transcription start site (TSS), transcription termination site (TTS), and translation start (ATG) and stop (TAA, TGA) codons are indicated. The direction of transcription is indicated by black arrows. *(A,C)* A gradual decrease of RNET-seq read coverage in the 5'-to-3' direction of *rho (A)* but not *prfB (C)* as displayed in IGV. Genome-aligned reads are in gray while mapped 3′ ends corresponding to paused RNAP are in blue and red in WT and dNusG cells, respectively. Numerous pause sites arose in *rho* in dNusG cells that were not observed in WT cells. *(B,D)* The fraction of 3' ends normalized to the total count of 3' ends in the region throughout *rho (B)* and *prfB (D)* in WT cells (blue) and dNusG cells (orange). Exponential fits of the data are marked by solid lanes and the corresponding equations are indicated. Upon NusG depletion, the decline in 3' end coverage toward the end of genes is increased for *rho* but decreased for *prfB* because of a strong pause site at the beginning of *prfB. (E)* NusG-suppressed pauses in *prfB* in WT and dNusG cells. Genome-aligned reads are in gray while mapped 3′ ends corresponding to paused RNAP are in blue and red in WT and dNusG cells, respectively. The values of $\log_2$ fold change in pause score after NusG depletion ($\log_2$FC) and the sequence around the pause site are indicated above and below the IGV panel, respectively. An in frame TGA stop codon that is a target for feedback regulation by the *prfB* gene product (termination factor RF2) is boxed and highlighted in gray. *(F)* Genome-wide normalized RNAP coverage along all annotated *E. coli* ORFs in WT and dNusG cells. The density of RNET-seq reads is plotted using a five-period moving average. NusG depletion does not impact transcription polarity.

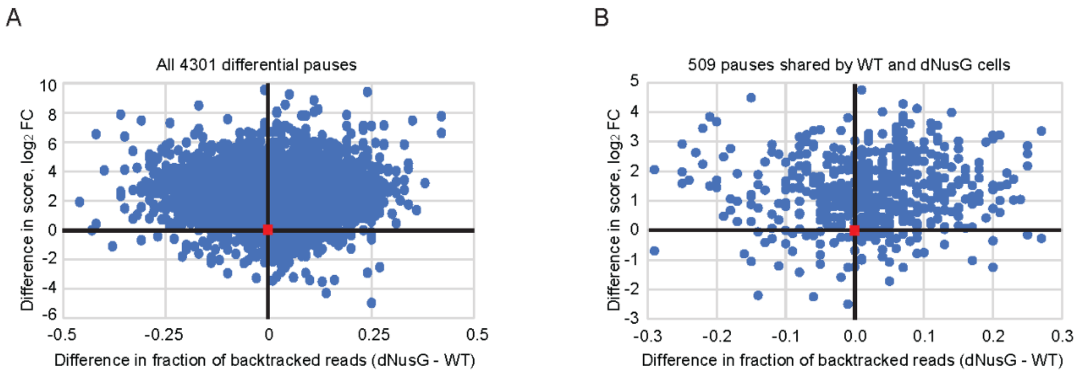

Fig. S14. Lack of correlation between changes in the fraction of backtracked reads and pause score after NusG depletion. *(A)* Correlation according to data of differential pause strength from *SI Appendix*, Dataset S5. Upon NusG depletion, the average increase in the fraction of backtracked reads was 0.01, whereas the average $\log_2$FC of the pause score was 2.5. The dots located at the left top, central top, and right top portions of this plot correspond to 3 groups of 50 NusG-suppressed pause sites shown in *SI Appendix*, Fig. S11, in which the fraction of backtracked reads was decreased, not affected, or increased by NusG depletion. *(B)* Correlation among pauses that were shared by WT and dNusG cells from *SI Appendix*, Dataset S3. After NusG depletion, the average increase in the fraction of backtracked reads was 0.02, whereas the average $\log_2$FC of the pause score was 1.3. (*A,B*) The red dot at the “0” coordinate of each plot indicates the position of the pause sites where backtracking and pause strength (measured as the fraction of >18 nt reads and the pause score, respectively) were not affected by NusG depletion. Notably, most of the pauses possess $\log_2$FC values above 0 (Y axis), indicating a global increase in the pause strength upon NusG depletion. The X-axis indicates a change in the fraction of >18 nt backtracked reads at each site upon NusG depletion. Apparently, the NusG-suppressed pauses showed no correlation with the abundance of the backtracked fraction at each pause as backtracking was decreased or increased at a similar number of pause sites upon NusG depletion. Moreover, the positive or negative effect of NusG depletion on backtracking did not exceed 20-25% (0.2-0.25 values of X-axis) for the majority of the pause sites.

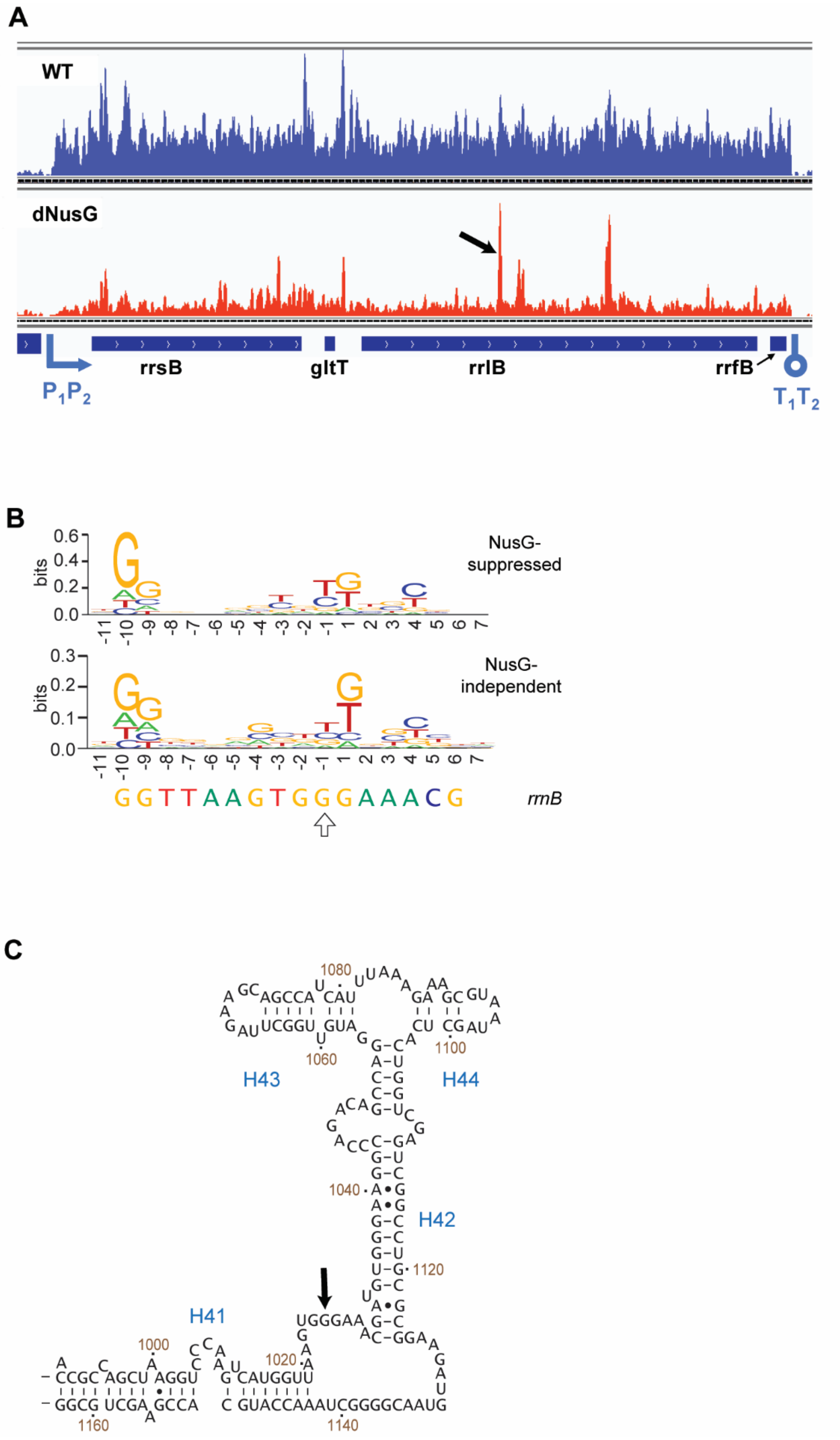
A
WT
dNusG
rrsB
gltT
rrlB
rrfB
P_1P_2
T_1T_2
B
bits
NusG-suppressed
NusG-independent
-11 -10 -9 -8 -7 -6 -5 -4 -3 -2 -1 1 2 3 4 5 6 7
GGTTAAGTGGGAAACG
rrnB
C
H41
H42
H43
H44
1000
1020
1040
1060
1080
1100
1120
1140
1160

Fig. S15. NusG depletion inhibits transcription of rRNA genes identified by RNET-seq. *(A)* Transcription traffic in the *rrnB* operon is reduced 2-fold after NusG depletion. Arrow marks the position of the strongest NusG-suppressed pause in the 23S rRNA gene. Positions of the promoters (P_1 and P_2) and terminators (T_1 and T_2) are shown *(B)* Comparison of the sequence logos of NusG-suppressed and NusG-independent pauses in protein-coding genes (Fig. 3*C,D*). The sequence of the strongest pause in the 23S rRNA gene of the *rrnB* operon is shown at the bottom. Position -1 corresponds to the 3' end of paused RNA and is marked with an arrow. *(C)* Secondary structure of a fragment of *E. coli* 23S rRNA that participates in binding incoming aa-tRNAs. 23S rRNA helices are marked by blue numbers (6). The 3' end of nascent RNA at the strongest NusG-suppressed pause site is indicated by an arrow.

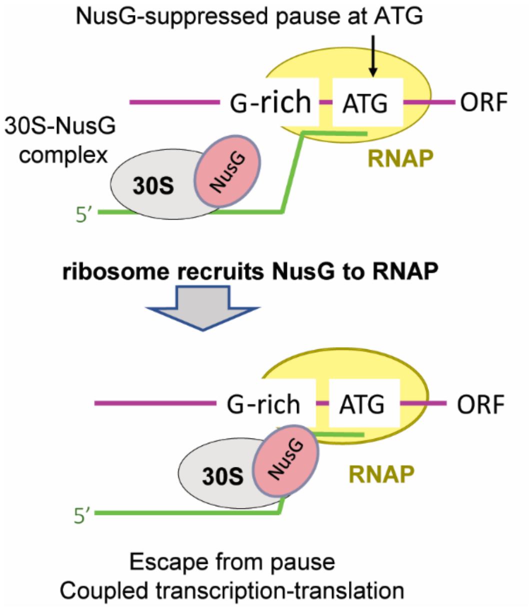

Fig. S16. Model of NusG-bridged RNAP-ribosome complexes at NusG-suppressed pause sites. RNAP pausing provides time for association of a NusG-bound 30S ribosomal subunit. The delivered NusG releases this pause at the translation initiation codon. Ribosome-assisted recruitment of NusG to RNAP results in the formation of a coupled transcription-translation complex containing RNAP and a 30S ribosomal subunit that is bridged by NusG. Binding of NusG-30S complex to RNAP signals escape from pausing at ATG start codons.

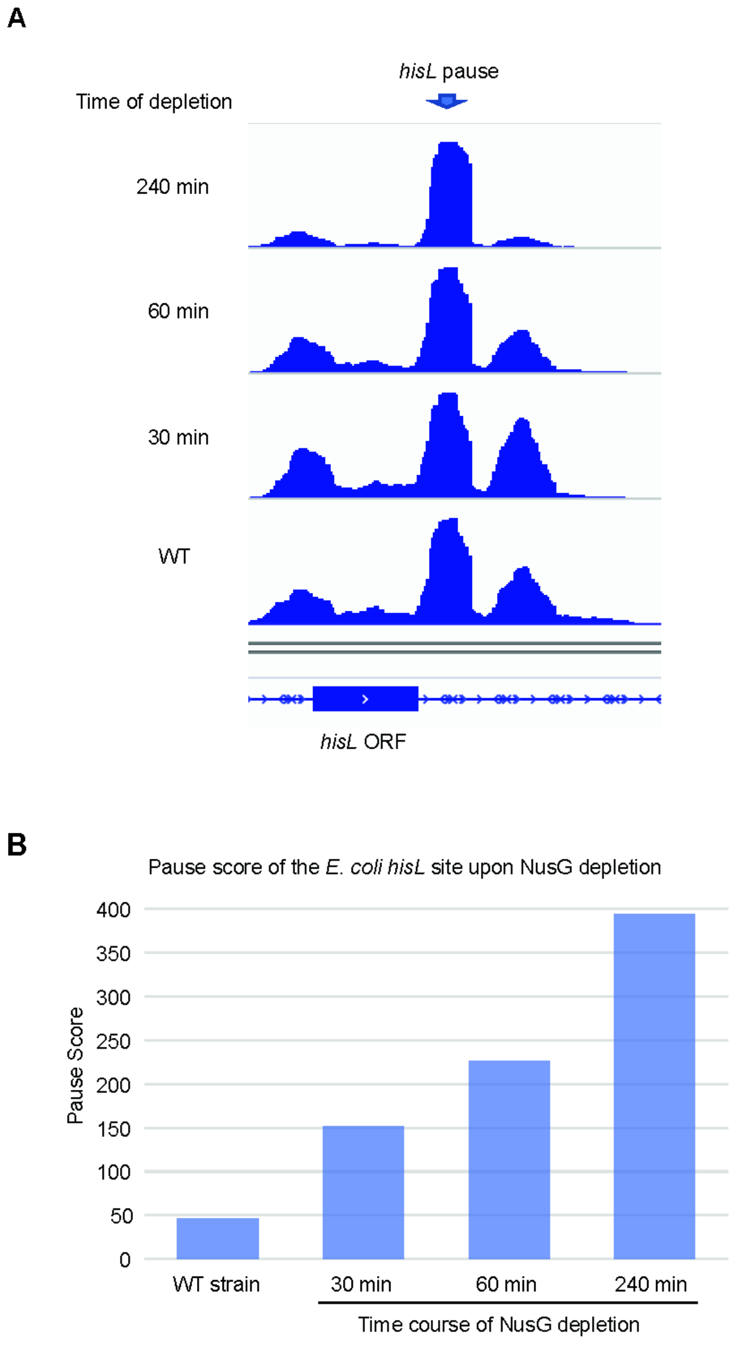

Fig. S17. Dynamics of pause strength at the *hisL* pause follows the time course of NusG depletion. *(A)* The dCas9 roadblock of *nusG* was induced with arabinose for 30, 60 and 240 min. The region around the *hisL* pause site upstream of the *hisG* ORF is shown as it appears in IGV. The autoscaled track height is normalized relative to the maximal value of the *hisL* pause. *(B)* Quantification of the results shown in panel A. The ±50 bp window surrounding the pause site used for quantification of pause score values for *hisL* was broader than the region shown in panel A.

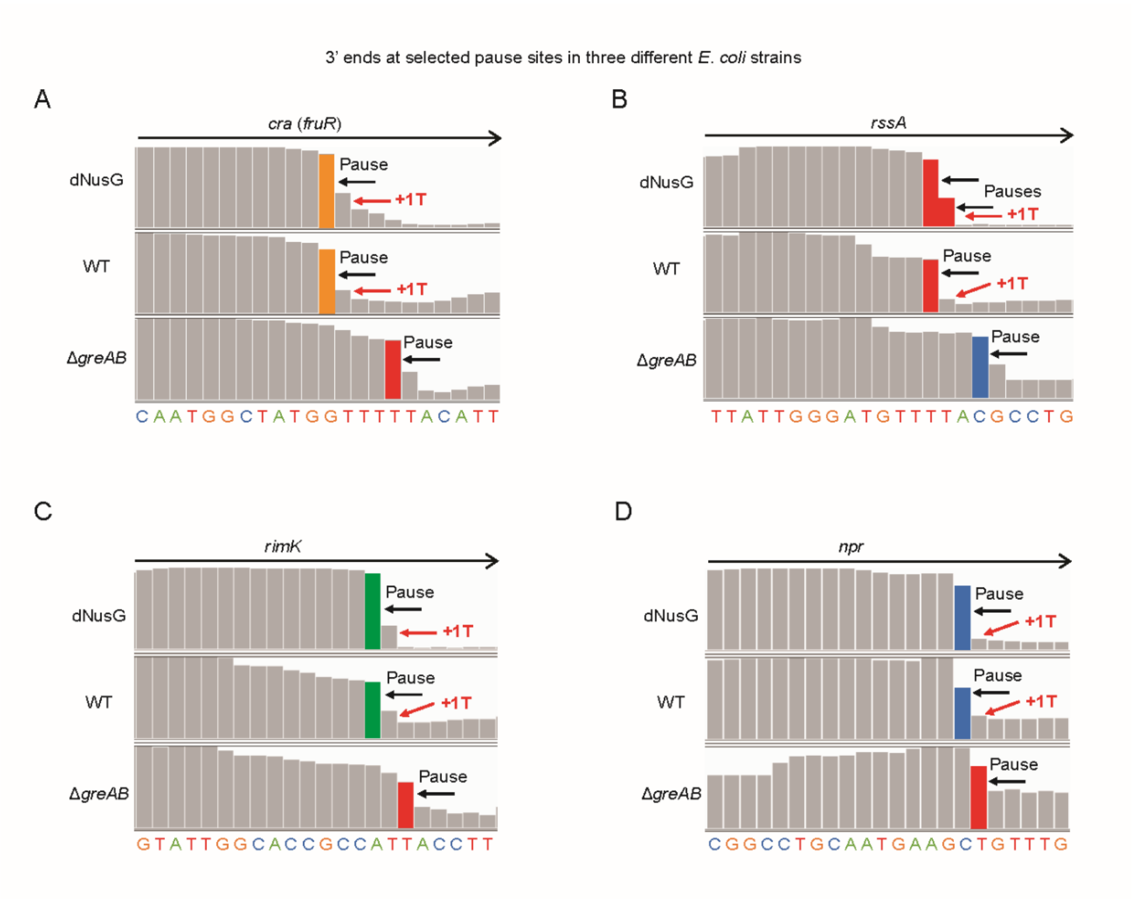

Fig. S18. *In vivo* cleavage of nascent RNA by Gre factors contributes to the appearance of the +1T residue in the sequence logo of pause sites. Genome-aligned reads are in gray while 3’ RNA ends at pauses from dNusG, WT (this work), and Δ*greA* Δ*greB* (9) *E. coli* strains are shown in color according to the identity of the bases at *cra* (*fruR*) *(A), rssA (B), rimK (C),* and *npr (D)* pause sites. Long black arrows indicate the direction of transcription. Sequence around the pause sites is indicated. Backtracking of elongation complexes is followed by cleavage of the nascent RNA by Gre factors upstream of a U residue in WT and dNusG cells (Fig. 4*D*). In turn, cleavage shifts the paused 3’ ends 1 to 4 bases upstream relative to -1Y 3’ ends in non-cleaving Δ*greA* Δ*greB* cells. Cleavage also results in the appearance of the +1T residue in the sequence logo of pauses in WT and dNusG cells.

Table S1. Summary of data from *SI Appendix*, Datasets 1 to 6.

				location [b]		+1 nucleotide [c]				length of sequence reads [d]				
	dataset	[a]	all	ORF	UTR	A	C	G	T	≤15	16	17	18	≥19
1	all WT	#	1613	891	722	236	272	505	600	204	135	370	433	470
	_50 [e]	%	100	55	45	15	17	31	37	13	8	23	27	29
1	all dNusG	#	5091	4149	942	584	419	2051	2037	502	392	991	1260	1947
	_50 [e]	%	100	81	19	11	8	40	40	10	8	19	25	38
2	merged	#	1057	602	455	134[g]	178	359	386	127 [g]	83	251	291	304
	WT [f]	%	100	57	43	13	17	34	37	12	8	24	28	29
2	merged	#	3753	3124	629	432[g]	288	1643	1390	332 [g]	265	720	944	1493
	dNusG [f]	%	100	83	17	12	8	44	37	9	7	19	25	40
3	WT	#	444	218	226	52	100	122	170	70	37	105	120	111
	only	%	100	49	51	12	23	27	38	16	8	24	27	25
3	dNusG	#	4408	3722	686	497	345	1781	1785	410	333	855	1101	1709
	only	%	100	84	16	11	8	40	40	9	8	19	25	39
3	WT	#	509	333	176	51	44	219	195	49	36	119	139	166
	shared	%	100	65	35	10	9	43	38	10	7	23	27	33
3	dNusG	#	509	333	176	51	44	219	195	70	43	102	117	177
	shared	%	100	65	35	10	9	43	38	14	9	20	23	35
3	WT	#	361	202	159	38	85	102	136	51	31	88	100	92
	single 3'	%	100	56	44	11	24	28	38	14	8	24	28	25
4	WT	#	17243	12281	4962	1960	3249	4986	7048	1801	1402	4212	4858	4966
	_12 [h]	%	100	71	29	11	19	29	41	10	8	24	28	29
4	dNusG	#	27449	22952	4497	3461	3100	10429	10459	2634	2120	5391	7051	10249
	_12 [h]	%	100	84	16	13	11	38	38	10	8	20	26	37
5	Differen	#	4301	3541	760	491	406	1761	1643	7 [k]	6	22	29	36
	tial_50 [i]	%	100	82	18	11	9	41	38	10 [l]	8	20	25	38
6	Differen	#	28171	23602	4569	3380	4076	9835	10880	8 [k]	7	23	29	34
	tial_12 [j]	%	100	84	16	12	14	35	39	11 [l]	8	20	25	35

[a] Number (#) or percent of total (%) of each kind of pause found in each dataset.
[b] Pauses within protein coding sequences (ORF) or intergenic (UTR).
[c] Count of each nucleotide at the +1 position located immediately downstream of the pause.
[d] Count of sequence reads that were ≤15, 16, 17, 18, or ≥19 nt in length.
[e] Pauses in wild-type cells (WT) and NusG-depleted cells (dNusG) identified using a stringent cut-off score ≥50.
[f] Pauses within a 10 bp window were merged into a single pause site and reported in *SI appendix,* Dataset S2.
[g] Nucleotide at the +1 position and length of sequence reads are reported for the highest score pause at pause sites with multiple 3' ends in Dataset S2.
[h] Pauses in WT and dNusG cells identified using less stringent cut-off score values ≥12.
[l] Differential pause strength identified using stringent cut-off of score values ≥50 in either WT or dNusG cells.
[j] Differential pause strength identified using less stringent cut-off of score values ≥12 in either WT or dNusG cells.

[k] Percent of total (%) of sequence reads that were ≤15, 16, 17, 18, or ≥19 nt in length in WT cells, highlighted in gray.
[l] Percent of total (%) of sequence reads that were ≤15, 16, 17, 18, or ≥19 nt in length in dNusG cells, highlighted in yellow.

Table S2. List of the strongest pauses. Less than a single pause site from this Table is predicted to occur per genome according to the negative exponential dependence between the number and the score of pauses (Fig. 1*C* and *SI Appendix*, Dataset S3).

A. Pauses with score values above 2200 in WT cells

gene	pause location	gene offset[a]	sense [b]	expr [c]	score	sequence [d]	+1 [e]	long [f]
fruB	2261575	-58	SENSE	3.4	3521	cctaagccagg**T**tggc	T	0.28
essD	576466	-155	SENSE	59	2895	ggttacttcgt**C**acac	A	0
ygaM	2798575	78	ANTI	6.5	2500	gtagggggatt**T**ttgt	T	0.52

B. Pauses with score values above 3200 in dNusG cells

gene	pause location	gene offset[a]	sense [b]	expr [c]	score	sequence [d]	+1 [e]	long [f]
dtd	4075095	0.13	SENSE	150	5614	agggagaagtg**A**cggg	C	0.51
glxR [g]	535768	-42	SENSE	1.5	5077	aggggatcgtg**T**tgtc	T	0.37
yfiL	2739301	-81	ANTI	7.1	4546	agggttgaaag**C**gcga	G	0.55
yihN	4060269	-1	SENSE	27	4310	ttaggggatgt**T**atgc	A	0.38
waaQ	3806207	-86	SENSE	39	4200	ggggcggtagc**G**tgct	T	0.4
yihN [g]	4060227	-43	SENSE	27	4081	tcggtatctta**T**tgcc	T	0.41
yghG	3110926	163	SENSE	63	3543	ctggctgatgc**T**gctt	G	0.48
fabA [g]	1015249	0.86	SENSE	104	3534	atggcgaagtg**C**tggt	T	0.5
leuL	83641	0.78	SENSE	2529	3494	cggtagacgag**T**gagc	G	0.36
tsaD [g]	3208439	0.12	SENSE	30	3494	acggcggcgtc**G**tgcc	T	0.36
dppA	3705690	0.02	SENSE	42	3488	atgctgaagct**T**ggtc	G	0.45

[a] Relative position of the pause within an ORF for the sites in coding regions (between 0.01 and 0.99), a negative integer indicates the distance in base pairs to the nearest downstream coding sequence for pauses in a 5' UTR, or a positive integer indicates distance to the nearest upstream coding sequence for pauses in a 3' UTR.
[b] Pause in sense or antisense strand.
[c] Expression in transcripts per kilobase million (TPM).
[d] Sequence surrounding the pause (3' RNA end at pause is capitalized).
[e] Nucleotide at the +1 position of the non-template strand immediately downstream of the pause.
[f] Fraction of long sequence reads (>18 nt in length) originated from backtracked elongation complexes.
[g] Highlighted in yellow are the pauses found only in dNusG cells. All other pauses are shared between WT and dNusG cells.

Table S3. Biological pathways with the largest increase in expression after NusG depletion.

A. RNA-seq data

Gene Ontology: Biological Process	GO [a]	Affected genes	Fold Enrichment [b]	FDR [c]
primary alcohol catabolic process	0034310	17[d]/22[e]	7.1	7.6E-06
ethanolamine-containing compound metabolic process	0042439	12/16	6.9	4.6E-04
ethanolamine metabolic process	0006580	12/16	6.9	4.3E-04
primary amino compound metabolic process	1901160	12/18	6.2	8.0E-04
primary alcohol metabolic process	0034308	19/33	5.3	2.6E-05
amine catabolic process	0009310	15/31	4.5	1.1E-03
pilus organization [f]	0043711	23/54	3.9	8.2E-05
alcohol catabolic process	0046164	19/46	3.8	6.2E-04
SOS response [f, g]	0009432	12/30	3.7	2.0E-02
organic hydroxy compound catabolic process	1901616	19/52	3.4	1.9E-03
monosaccharide transmembrane transport	0015749	14/42	3.1	3.0E-02

B. RNET-seq data

Gene Ontology: Biological Process	GO [a]	Affected genes	Fold Enrichment [b]	FDR [c]
SOS response [f, g]	0009432	9[d]/30[e]	5.4	2.0E-02
cell adhesion involved in single-species biofilm formation	0043709	11/42	4.7	1.2E-02
pilus organization [f]	0043711	14/54	4.6	2.9E-03
single-species biofilm formation	0044010	19/74	4.6	1.6E-04
single-species submerged biofilm formation	0090609	13/51	4.6	5.4E-03
biofilm formation	0042710	23/98	4.2	8.4E-05
cell-substrate adhesion	0031589	11/47	4.2	2.2E-02
submerged biofilm formation	0090605	13/56	4.1	8.2E-03
biological adhesion	0022610	12/62	3.5	3.7E-02
cell projection organization	0030030	14/81	3.1	3.6E-02

[a] Gene Ontology number.
[b] Ratio of observed to expected gene numbers.
[c] False discovery rate.
[d] Number of recovered genes.
[e] Total number of genes in a process.
[f] Biological processes affected in both RNA-seq and RNET-seq expression data.
[g] NusG involvement in this biological process (highlighted in yellow) is explained in the text.

Table S4. Biological pathways with the largest decrease in expression after NusG depletion.

A. RNA-seq data

Gene Ontology: Biological Process	GO [a]	Affected genes	Fold Enrichment [b]	FDR [c]
maltodextrin transport [f]	0042956	5[d]/5[e]	19	1.1E-02
L-arginine transmembrane transport	1903400	4/4	19	3.3E-02
L-arginine transport	1902023	4/5	15	4.8E-02
maltose transport [f]	0015768	5/7	14	2.4E-02
bacterial-type flagellum-dependent swarming motility	0071978	8/15	10	2.2E-03
polysaccharide transport [f]	0015774	5/10	9.6	4.7E-02
ribosomal small subunit assembly [g]	0000028	7/20	6.7	3.0E-02
ribosome assembly [g]	0042255	17/55	5.9	4.6E-05
ribonucleoprotein complex assembly [g]	0022618	15/49	5.9	1.6E-04
ribosomal large subunit biogenesis [g]	0042273	8/29	5.3	3.1E-02
bacterial-type flagellum-dependent cell motility	0071973	10/41	4.7	2.5E-02
non-membrane-bounded organelle assembly	0140694	18/74	4.7	1.7E-04
cilium or flagellum-dependent cell motility	0001539	10/44	4.4	2.8E-02
cellular protein-containing complex assembly	0034622	16/73	4.2	1.7E-03
cell motility	0048870	11/51	4.1	2.4E-02
translation [g]	0006412	25/118	4.1	3.1E-05
peptide biosynthetic process [g]	0043043	27/130	4.0	2.3E-05
movement of cell or subcellular component	0006928	12/59	3.9	2.6E-02
locomotion	0040011	12/65	3.5	3.3E-02
peptide metabolic process	0006518	27/148	3.5	6.3E-05
ribosome biogenesis [g]	0042254	17/100	3.3	1.1E-02
ribonucleoprotein complex biogenesis [g]	0022613	17/101	3.2	1.1E-02
organelle organization	0006996	21/125	3.2	2.2E-03
amide biosynthetic process	0043604	27/165	3.1	2.0E-04

B. RNET-seq data

Gene Ontology: Biological Process	GO [a]	Affected genes	Fold Enrichment [b]	FDR [c]
maltodextrin transport [f]	0042956	5[d]/5[e]	> 100	6.6E-06
maltose transport [f]	0015768	5/7	> 100	1.0E-05
polysaccharide transport [f]	0015774	5/10	76	2.6E-05
disaccharide transport	0015766	5/16	47	1.0E-04
oligosaccharide transport	0015772	5/19	40	1.7E-04
carbohydrate transport	0008643	7/126	8.4	6.8E-03

[a] Gene Ontology number.
[b] Ratio of observed to expected gene numbers.
[c] False discovery rate.

[d] Number of recovered genes.
[e] Total number of genes in a process.
[f] Biological processes affected in both RNA-seq and RNET-seq expression data.
[g] NusG involvement in these biological processes (highlighted in yellow) is explained in the text.

Table S5. Predicted pause hairpins for pause sites that were characterized *in vitro*. Transcribed DNA sequences predicted to fold into pause hairpins in nascent RNA are underlined. Yellow highlights indicate the sequences of the antisense oligonucleotides that were used to interfere with the hairpin folding *in vitro*. 3' ends of pauses identified *in vivo* are marked in bold red. 3' ends of pauses identified *in vitro* but not observed *in vivo* are marked in bold blue.

Pause Site	Upstream Sequence	ΔG [a]
hisL	CCATCATCACCATCATCCTGACTAGTCTTTCAGGCGATGTGTGC**T**GGAAGACA	-5.0
hslU	CCACACCATCGAAGAATTAAGCTACAAAGCGTAAGGATCTCCC**C**A**T**GTCTGAAATG	-2.3
ibpB	CCGTCAGGGAGCATATGCGAATCTTCGGATTTGCAGGTACTTACT**C**GCTTCTTAG	-12.4
yajD	CCAACCTGCGTGAACTTACCGTTCACCACATTGATCA**C**GACCATACCAA	-7.3

[a] Free energy of hairpin folding, kcal/mol.

Table S6. Among pairs of two adjacent pauses, the downstream pauses (n+1 rows) generally possess a higher fraction of long backtracked (>18 nt) reads than the upstream pauses (n rows).

gene	pause location	DNA [a]	gene offset [b]	$\log_2$ FC [c]	Δ FC [d]	sequence [e]	Fraction of read length [f] <16	16	17	18	>18	Δ [g] long
ccmA	2295508	$-^{n}$	0.25	0.92		tgacgggttgtcTcgcc	0.04	0.03	0.35	0.33	0.24	
ccmA	2295507	$-^{n+1}$	0.26	0.8	-0.1	gacgggttgtctCgccc	0.05	0.03	0.06	0.33	0.54	0.30
cusA	600627	$+^{n}$	0.86	0.85		gggtggcgaagcGttgc	0.03	0.08	0.3	0.33	0.26	
cusA	600628	$+^{n+1}$	0.86	0.59	-0.3	ggtggcgaagcgTtgct	0.03	0.07	0.18	0.23	0.5	0.24
dtd	4075094	$+^{n}$	0.13	3.0		tggaggagaagtGacgg	0.06	0.11	0.26	0.29	0.29	
dtd	4075095	$+^{n+1}$	0.13	3.6	0.6	ggaggagaagtgAcggg	0.04	0.04	0.16	0.24	0.51	0.22
fabA	1015250	$-^{n}$	0.86	4.8		cggatggcgaagtGctgg	0.03	0.08	0.28	0.26	0.35	
fabA	1015249	$-^{n+1}$	0.86	6.1	1.3	ggatggcgaagtgCtggt	0.01	0.04	0.17	0.27	0.5	0.15
holB	1155861	$+^{n}$	0.87	3.5		tatactgggggatGtttg	0.11	0.03	0.26	0.42	0.19	
holB	1155862	$+^{n+1}$	0.87	5.9	2.4	atactgggggatgTttgc	0.09	0.03	0.06	0.33	0.49	0.30
otsB	1980028	$-^{n}$	0.48	2.8		cgaaagggatggcTtttg	0.05	0.07	0.27	0.29	0.32	
otsB	1980027	$-^{n+1}$	0.48	4.4	1.6	gaaagggatggctTttgc	0.03	0.03	0.2	0.27	0.46	0.14
rarA	937336	$+^{n}$	0.09	3.1		ctgcgggaagccGttgc	0.03	0.04	0.15	0.17	0.61	
rarA	937337	$+^{n+1}$	0.09	3.4	0.3	tgcgggaagccgTtgcc	0.01	0.03	0.07	0.16	0.72	0.11
rssA	1288678	$+^{n}$	0.23	2.5		ttattgggatgttTtacg	0.2	0.21	0.24	0.14	0.2	
rssA	1288679	$+^{n+1}$	0.23	4.9	2.4	tattgggatgtttTacgc	0.17	0.02	0.25	0.23	0.34	0.14
waaG	3804051	$-^{n}$	0.92	3.9		gcctggcggagaAtgct	0.1	0.15	0.36	0.22	0.17	
waaG	3804050	$-^{n+1}$	0.93	4.9	1.0	cctggcggagaaTgctc	0.05	0.03	0.11	0.4	0.42	0.25
waaQ	3806207	$-^{n}$	-86	nd [h]		ctggggcggtagcGtgct	0.04	0.08	0.24	0.23	0.4	
waaQ	3806206	$-^{n+1}$	-85	nd		tggggcggtagcgTgctt	0.01	0.04	0.12	0.23	0.59	0.19
ybaO	467605	$+^{n}$	-2	1.3		ctctgtggaagggCtatg	0.1	0.12	0.35	0.33	0.11	
ybaO	467606	$+^{n+1}$	-1	2.1	0.8	tctgtggaagggcTatgt	0.08	0.03	0.24	0.33	0.32	0.21
ybaP	507146	$-^{n}$	0.2	2.0		ccctgcccacccgTttgc	0.05	0.03	0.12	0.11	0.69	
ybaP	507145	$-^{n+1}$	0.2	1.0	-1.0	cctgcccacccgtTtgct	0.04	0.01	0.1	0.15	0.69	0
ybeU	679325	$+^{n}$	0.84	0.74		cgccggagtcgtAtgtt	0.03	0.04	0.18	0.24	0.52	
ybeU	679326	$+^{n+1}$	0.84	2.0	1.3	gccggagtcgtaTgttc	0.06	0.15	0.29	0.32	0.18	-0.34
ygaC	2797915	$-^{n}$	0.29	nd		gccgtggggaaaaTtatg	0.07	0.11	0.25	0.22	0.34	
ygaC	2797914	$-^{n+1}$	0.3	nd		ccgtggggaaaatTatgt	0.03	0.03	0.13	0.26	0.55	0.21
ygiC	3178732	$+^{n}$	0.25	6.2		caccagccatcgcTttat	0.02	0.03	0.13	0.23	0.58	
ygiC	3178733	$+^{n+1}$	0.25	6.3	0.1	accagccatcgctTtatt	0.04	0.06	0.28	0.15	0.47	-0.11
yiaA	3724949	$+^{n}$	1	3.3		gcttacgtgagttAttcg	0.05	0.05	0.09	0.14	0.67	
yiaA	3724950	$+^{n+1}$	0.99	3.4	0.1	cttacgtgagttaTtcgc	0.11	0.02	0.07	0.13	0.67	0
yihU	4071467	$+^{n}$	0.14	0.66		gggagtcgcacctTtgtc	0.04	0.05	0.19	0.23	0.5	
yihU	4071468	$+^{n+1}$	0.14	0.44	-0.2	ggagtcgcaccttTgtct	0.02	0.02	0.08	0.19	0.69	0.19
yjdJ	4350310	$-^{n}$	0.74	0.85		gcataatgggataAtttt	0.09	0.11	0.32	0.37	0.11	
yjdJ	4350309	$-^{n+1}$	0.74	1.63	0.8	cataatgggataaTtttt	0.15	0.03	0.13	0.3	0.39	0.28
ypjD	2746309	$+^{n}$	0.41	3.9		gcgctggcaacctTcatg	0.01	0.16	0.34	0.18	0.3	
ypjD	2746310	$+^{n+1}$	0.41	5.5	1.6	cgctggcaaccttCatgc	0.02	0.03	0.2	0.32	0.44	0.14

Δ Fraction = Sum (Fraction $_{n+1}$ - Fraction $_{n}$) [i]	**-0.1**	**-0.8**	**-1.8**	**0.1**	**2.6**

[a] Region is transcribed from + or - DNA strand according to the *E. coli* reference genome NC_000913.2.
[b] Relative position of the pause within an ORF (between 0.01 and 0.99). A negative integer indicates the distance to the nearest downstream protein-coding sequence for pauses in a 5' UTR. A positive integer indicates distance to the nearest upstream protein-coding sequence for pauses in a 3' UTR.
[c] The $\log_2$(Fold Change) of ratio of pause scores normalized to transcripts per kilobase million (TPM) values in dNusG vs. WT cells. nd, indicates the pauses that were not identified using differential pause strength analysis (*SI Appendix*, Datasets S5 and S6).
[d] Difference between Log_2FC values at the downstream pause (n+1 rows) and the upstream pause (n rows). Dominating positive values in this column indicate that the downstream pauses generally possess a larger fraction of backtracked reads than the upstream pauses among pairs of two adjacent pauses.
[e] Sequences are aligned relative to the 3' end of the paused RNA (marked with a capital letter in bold font). -9G +1T sequence at upstream pauses (n rows) are highlighted in cyan. -10G -1Y +1G sequence at downstream pauses (n+1 rows) are highlighted in yellow.
[f] Fraction of sequence reads that were <16, 16, 17, 18, or >18nt in length.
[g] Fraction of long reads (>18 nt) at downstream pauses (n+1 rows) minus fraction of long reads at upstream pauses (n rows).
[h] nd, not determined by our bioinformatic pipeline.
[i] Sum of the differences between the fraction at each downstream pause and each upstream pause (n+1 row) – (n row) for each read length.

Table S7. ***In vitro*** **characterization of NusG-affected pause sites identified** ***in vivo*****.**

pause site	DNA [b]	log_2FC -TPM [c]	genome position [a] WT and dNusG	ΔgreAB	in vitro	sequence [d]	in vitro $T_{1/2}$ (s) [e] -	NusG
coaA	-	-3.5	4173069	4173065	4173069<br>4173065[f]	cgatagagctaTgaccgcc	11	7
dppA	-	5.3	3705690	same	same	atgctgaagctTggtctca	24	9
dtd	+	3.9	4075095	4075096	4075096	agggagaagtgAcgggcga	116	69
frmB	-	-6.3	377606	same	same	gggattaacccTgagataa	16	7
gltB	+	4.6	3352688	same	same	cgctctttctgTgcccgtg	87	13
hisL	+	2.3	2088090	same	same	gcgatgtgtgcTggaagac	63	29
hslU	-	5.7	4119771	4119769	4119769	taaggatctccCatgtctg	26	17
ibpB	-	-5.0	3864944	same	same	aggtacttactCgcttctt	120	110
leuL	-	3.6	83641	same	same	cggtagacgagTgagcggc	28	13
nuoA	-	7.9	2402712	same	same	ctggacgcccgCgcgttca	25	9
uup	+	5.2	1009187	1009188	1009188	aaggaatagtaAtgtcatt	43	15
waaQ (ops)	-	nd [g]	3806207<br>3806206	same	3806208<br>3806206[h]	ggggcggtagcGTgctttt	39	19
yajD	+	10.4	429975	same	same	cacattgatcaCgaccata	23	18 [i]
yghG	-	1.7	3110926	same	same	ctggctgatgcTgcttatt	25	13

[a] Genomic coordinate (NC_000913.2) of NusG-affected pauses *in vitro* and *in vivo* in WT, dNusG, and *greAB E. coli* cells (this work and ref. 9).
[b] Region is transcribed from the + or - DNA strand according to the *E. coli* reference genome NC_000913.2.
[c] From *SI Appendix*, Dataset S5.
[d] Sequences surrounding the *in vivo* pauses were aligned relative to the 3' ends (marked with capital letter and bold font). 3' end positions of *in vitro* pauses are shown in red and the adjacent +1G residue (5' of the pause) is highlighted in yellow. -10G residues of the *in vitro* and the *in vivo* pauses are highlighted in cyan.
[e] *In vitro* pause half-life ± NusG.
[f] Only one of the two indicated pauses in *coaA* was found *in vivo* in WT, dNusG or Δ*greA* Δ*greB* cells. Both pauses were observed *in vitro*. Pause half-lives are indicated for position C4173065.
[g] nd, not determined by our bioinformatic pipeline.
[h] Pause half-lives are indicated for position T3806206.
[i] NusG decreases the pausing efficiency from 26% to 4% (Fig. 2).

Table S8. ***E. coli*** **strains used in this study.**

Strain	Genotype [a]	Source
SJ_XTL219	MG1655 *galM*<pBBa-J23119-Tc^{r}-*sacB*-handle-(*S. pyogenes* terminator)-(*rrnB* terminator)>*gmpA*; pSIM18	(2)
NB854	W3110 *rpoC*-6xHis-Km^{r} *gal490*	(8)
NB959	W3110 *rpoC*-6xHis-Km^{r} Δ*greA* Tc^{r} Δ*greB* Ap^{r}	(8)
NB1246	SJ_XTL219 *rpoC*-6xHis-Km^{r}	this study
NB1247	NB1246 dcas9/sgRNA-*nusG*	this study

[a] Km, kanamycin; Tc, tetracycline; Ap, Ampicillin

Table S9. Plasmids used in this study.

Plasmid	Description [a]	Source
pSIM18	pSC101^{ts} Hy^{r}, λ red DNA recombineering plasmid	(2)
pTZ19R	cloning vector Ap^{r}	Thermo Fisher Scientific
pAY196	P_{trp} promoter and *tlrB* leader in pTZ19R	(18)

[a] Hy, hygromycin; Ap, ampicillin

Table S10. Sequence of DNA and RNA oligonucleotides used in this study.

Oligo nucleotide	Application	Sequence
sgRNA-*nusG*	sgRNA-*nusG* construction	TTGACAGCTAGCTCAGTCCTAGGTATAATGCTAGCACGACGTACC AGCGCTTTTTGTTTTAGAGCTAGAAATAGCAAGTTAAAATAAGGC
galM-F-check	sgRNA-*nusG* confirmation	AGCCGTTATACCTTTGACGGTG
sgRNA-*nusG*-R-check	sgRNA-*nusG* confirmation	CAAAAAGCGCTGGTACGTCGTG
barcode DNA linker	ligation to RNA 3'end	rAppNNNNNNCTGTAGGCACCATCAAT/3ddC
oRTay7	reverse transcription	/5Phos/GATCGGAAGAGCACACGTCTGAACTCCAGTCAC/iSp18/CA CTCA/iSp18/TCCGACGATCATTGATGGTGCCTACAG [a]
oLSC006	library sequencing	TCCGACGATCATTGATGGTGCCTACAG
oI2ayRead	index 2 (i5) reading	GGCACCATCAATGATCGTCGGAGTGT
oGAB11	control for ligation and RT reactions	rArGrUrCrArCrUrUrArGrCrGrArUrGrUrArCrArCrUrGrArCrUrGrUrG
(01 to 12) o'i7	reverse indexing	CAAGCAGAAGACGGCATACGAGAT['i7]GTGACTGGAGTTCAGAC GTGTGCTCTTCCGATC [b]
(1 to 8) i5AY	forward indexing	AATGATACGGCGACCACCGAGATCTACAC[i5]ACACTCCGACGAT CATTGATGGTGCCTACAG [c]
AS_hisL	antisense	CTAGTCAGGATGATGG
AS_hslU	antisense	GCTTAATTCTTCGATGG
AS_ibpB	antisense	CCGAAGATTCGCATAT
AS_yajD	antisense	GGTAAGTTCACGCAGG
PSL	txn [d]	CAGCTTGACAAATACACAAGAGTGTGTTATAATGCAATTAG
frmB Fr	txn	CCCTCGAGTTCCTGGCCTCTCCCCGGGCAGCCGTC
frmB Bk	txn	GCTGACATGTTTTTCAATGAGTTCC
gltB Fr	txn	CCCTCGAGTTCCTGGCCGGAGGCGCGCGTATGACAC
gltB Bk	txn	CTGCGGGAACCCCACTTCC
hisL Fr	txn	CCCTCGAGTTCCTGGCCATCATCACCATCATCCTGAC
hisL Bk	txn	CATGCGTTCATGCACCACTGG
hslU Fr	txn	CCCTCGAGTTCCTGGCCACACCATCGAAGAATTAAGCTAC
hslU Bk	txn	CGCTGACGATTTCGCGTGG
ibpB Fr	txn	CCCTCGAGTTCCTGGCCGTCAGGGAGCATATGCG
ibpB Bk	txn	CGAAGTTACGCATAGTCATTTCTCC
nuoA Fr	txn	CCCTCGAGTTCCTGGCCTGGTGCGTATTGGCGCGC
nuoA Bk	txn	CGTTTCCGGGTTCATACGCTC
uup Fr	txn	CCCTCGAGTTCCTGGCCACAACTGCTGGCTGATTACC
uup Bk	txn	CGACAGCCATGCGCCATGC
yajD Fr	txn	CCCTCGAGTTCCTGGCCAACCTGCGTGAACTTACCG
yajD Bk	txn	CCCAGTTACTGCCATCTTCCG
waaQ Fr	txn	CCCTCGAGTTCCTGGCCAGGTAGCTGTTGAGCCTGG
waaQ Bk	txn	GCGACTCTTTTGTGTGATTGTC
yghG Fr	txn	CCCTCGAGTTCCTGGCCTGCGAAAAATTGCACGCGGG
yghG Bk	txn	GCGCCAGAGTGAATGCGCC
leuL Fr	txn	CCCTCGAGTTCCTGGCCTAAACGCATCTTCTTTGCGCGG
leuL Bk	txn	GTTTGACTGCGTGCTGGC
dtd Fr	txn	CCCTCGAGTTCCTGGCCCGTGCCAGCGTCACCGTG
dtd Bk	txn	ACACCAAAAGTCCCGCGCC

196 Bk	txn	GGCCAGGAACTCGAGGG

[a] iSp18 is a 18-carbon space.
[b] O1'i7 to 12'i7 are used for barcoding on the Illumina platform. Sequences [1'i7] to [12'i7] are the reverse complement of the Illumina barcodes D701 to D712, respectively, that are read during sequencing. For example, the sequence of [1'i7] is CGAGTAAT.
[c] 1i5AY to 8i5AY are used for barcoding on the Illumina platform. Sequences [1i5AY] to [8i5AY] are the Illumina barcodes D501 to D508, respectively, that are read during sequencing. For example, the sequence of [1i5AY] is TATAGCCT.
[d] PCR amplification of templates for *in vitro* transcription.

Dataset S1 (separate file). Pauses identified by RNET-seq in WT or dNusG cells that have score values at least 50 and contain at least 533 RNET-seq reads.

Dataset S2 (separate file). Multiple adjacent pauses from Dataset S1 located within a 10 bp window were merged into a single pause site.

Dataset S3 (separate file). Pauses with score values at least 50 that are unique or shared by WT or dNusG cells.

Dataset S4 (separate file). Pauses in WT or dNusG cells that have score values at least 12 and contain at least 200 sequencing reads.

Dataset S5 (separate file). Differential strength of pauses that have score values ≥50 in either WT or dNusG cells.

Dataset S6 (separate file). Differential strength of pauses that have score values >12 in either WT or dNusG cells.

Dataset S7 (separate file). Differential gene expression in WT and dNusG cells based on RNET-seq and RNA-seq data. False Discovery Rate (FDR) cutoff of 0.05 was used. The Rac prophage and *nusG* genes repressed in dNusG cells are highlighted in yellow and not used for the analysis of differential gene expression.

Dataset S8 (separate file). The strongest NusG-suppressed pauses that possess positive, negative, or no correlation between effect of NusG depletion on pausing strength and backtracking.

Dataset S9 (separate file). CLC Genomics Workbench 21.0.5 software (Qiagen) predicts RNA hairpins upstream from 458 pause sites that exhibit the >300 score values in dNusG cells and the strongest pause suppression by NusG (log_2FC-Pause-TPM >3).

SI References

1. M. Bubunenko, T. Baker, and D.L. Court, Essentiality of ribosomal and transcription antitermination proteins analyzed by systematic gene replacement in *Escherichia coli. J. Bacteriol.* 189, 2844-2853 (2007).
2. X.T. Li, Y. Jun, M.J. Erickstad, S.D. Brown, A. Parks, D.L. Court, S. Jun, tCRISPRi: tunable and reversible, one-step control of gene expression. *Sci. Rep.* 6, 39076 (2016).
3. A.V. Yakhnin, P.C. FitzGerald, C. McIntosh, H. Yakhnin, M. Kireeva, J. Turek-Herman, Z.F. Mandell, M. Kashlev, P. Babitzke, NusG controls transcription pausing and RNA polymerase translocation throughout the *Bacillus subtilis* genome. *Proc. Natl. Acad. Sci. U.S.A.* 117, 21628-21636 (2020).
4. R. Landick, D. Wang, C.L. Chan, Quantitative analysis of transcriptional pausing by *Escherichia coli* RNA polymerase: his leader pause site as paradigm. *Methods Enzymol.* 274, 334-353 (1996).
5. C.L. Squires, J. Greenblatt, J. Li, C. Condon, C.L. Squires, Ribosomal RNA antitermination in vitro: requirement for Nus factors and one or more unidentified cellular components. *Proc. Natl. Acad. Sci. U.S.A.* 90, 970-974 (1993).
6. A.S. Petrov, C.R. Bernier, E. Hershkovits, Y. Xue, C.C. Waterbury, C. Hsiao, V.G. Stepanov, E.A. Gaucher, M.A. Grover, S.C. Harvey, N.V. Hud, R.M. Wartell, G.E. Fox, L.D. Williams, Secondary structure and domain architecture of the 23S and 5S rRNAs. *Nucleic Acids Res.* 41, 7522-7535 (2013).

7. S. Borukhov, V. Sagitov, A. Goldfarb, Transcript cleavage factors from *E. coli. Cell* 72, 459-66 (1993).
8. M. Imashimizu, H. Takahashi, T. Oshima, C. McIntosh, M. Bubunenko, D. L. Court, M. Kashlev, Visualizing translocation dynamics and nascent transcript errors in paused RNA polymerases in vivo. *Genome Biol.* 16, 98 (2015).
9. Z. Sun, A.V. Yakhnin, P.C. FitzGerald, C.E. McIntosh, M. Kashlev, Nascent RNA sequencing identifies a widespread sigma70-dependent pausing regulated by Gre factors in bacteria. *Nat. Commun.* 12, 906 (2021).
10. M. H. Larson, R. A. Mooney, J. M. Peters, T. Windgassen, D. Nayak, C. A. Gross, S. M. Block, W. J. Greenleaf, R. Landick, J. S. Weissman, A pause sequence enriched at translation start sites drives transcription dynamics in vivo. *Science* 344, 1042-1047 (2014).
11. I. O. Vvedenskaya, H. Vahedian-Movahed, J. G. Bird, J. G. Knoblauch, S. R. Goldman, Y. Zhang, R. H. Ebright, B. E. Nickels, Interactions between RNA polymerase and the "core recognition element" counteract pausing. *Science* 344, 1285-1289 (2014).
12. B. M. Burmann, K. Schweimer, X. Luo, M. C. Wahl, B. L. Stitt, M. E. Gottesman, P. Rösch, A NusE:NusG complex links transcription and translation. *Science* 328, 501-504 (2010).
13. E.J. Bailey, M.E. Gottesman, R.L. Gonzalez Jr, NusG-mediated coupling of transcription and translation enhances gene expression by suppressing RNA polymerase backtracking. *J. Mol. Biol.* 434, 167330 (2022).
14. R.A. Mooney, S.E. Davis, J.M. Peters, J.L. Rowland, A.Z. Ansari, R. Landick, Regulator trafficking on bacterial transcription units in vivo. *Mol. Cell* 33, 97-108 (2009).
15. W. Chan, N. Costantino, R. Li, S.C. Lee, Q. Su, D. Melvin, D.L. Court, P. Liu, A recombineering based approach for high-throughput conditional knockout targeting vector construction. *Nucleic Acids Res.* 35, e64 (2007).
16. J.A. Sawitzke, L.C. Thomason, M. Bubunenko, X. Li, N. Costantino, D.L. Court, Recombineering: highly efficient in vivo genetic engineering using single-strand oligos. *Methods Enzymol.* 533, 157-177 (2013).
17. A. V. Yakhnin, H. Yakhnin, P. Babitzke, RNA polymerase pausing regulates translation initiation by providing additional time for TRAP-RNA interaction. *Mol. Cell* 24, 547-557 (2006).
18. Z.F. Mandell, R.T. Oshiro, A.V. Yakhnin, R. Vishwakarma, M. Kashlev, D.B. Kearns, P. Babitzke, NusG is an intrinsic transcription termination factor that stimulates motility and coordinates gene expression with NusA. *Elife* 10, e61880 (2021).
19. J. R. Knowlton, M. Bubunenko, M. Andrykovitch, W. Guo, K.M. Routzahn, D.S. Waugh, D.L. Court, X. Ji, A spring-loaded state of NusG in its functional cycle is suggested by X-ray crystallography and supported by site-directed mutants. *Biochemistry* 42, 2275-2281 (2003).
20. I. Artsimovitch, R. Landick, The transcriptional regulator RfaH stimulates RNA chain synthesis after recruitment to elongation complexes by the exposed nontemplate DNA strand. *Cell* 109, 193-203 (2002).
